# Characterizing Neonatal Heart Maturation, Regeneration, and Scar Resolution Using Spatial Transcriptomics

**DOI:** 10.3390/jcdd9010001

**Published:** 2021-12-21

**Authors:** Adwiteeya Misra, Cameron D. Baker, Elizabeth M. Pritchett, Kimberly N. Burgos Villar, John M. Ashton, Eric M. Small

**Affiliations:** 1 Department of Medicine, Aab Cardiovascular Research Institute, School of Medicine and Dentistry, University of Rochester, Rochester, NY 14642, USA; Adwiteeya_misra@urmc.rochester.edu (A.M.); kimberly_burgosvillar@urmc.rochester.edu (K.N.B.V.); 2 Department of Biomedical Engineering, University of Rochester, Rochester, NY 14642, USA; 3 Genomics Research Center, School of Medicine and Dentistry, University of Rochester, Rochester, NY 14642, USA; cameron_baker@urmc.rochester.edu (C.D.B.); Elizabeth_pritchett@urmc.rochester.edu (E.M.P.); john_ashton@urmc.rochester.edu (J.M.A.); 4 Department of Pathology, School of Medicine and Dentistry, University of Rochester, Rochester, NY 14642, USA; 5 Department of Pharmacology and Physiology, University of Rochester, Rochester, NY 14642, USA

**Keywords:** heart, regeneration, scar, mouse, fibroblast, spatial transcriptomics

## Abstract

The neonatal mammalian heart exhibits a remarkable regenerative potential, which includes fibrotic scar resolution and the generation of new cardiomyocytes. To investigate the mechanisms facilitating heart repair after apical resection in neonatal mice, we conducted bulk and spatial transcriptomic analyses at regenerative and non-regenerative timepoints. Importantly, spatial transcriptomics provided near single-cell resolution, revealing distinct domains of atrial and ventricular myocardium that exhibit dynamic phenotypic alterations during postnatal heart maturation. Spatial transcriptomics also defined the cardiac scar, which transitions from a proliferative to secretory phenotype as the heart loses regenerative potential. The resolving scar is characterized by spatially and temporally restricted programs of inflammation, epicardium expansion and extracellular matrix production, metabolic reprogramming, lipogenic scar extrusion, and cardiomyocyte restoration. Finally, this study revealed the emergence of a regenerative border zone defined by immature cardiomyocyte markers and the robust expression of *Sprr1a.* Taken together, our study defines the spatially and temporally restricted gene programs that underlie neonatal heart regeneration and provides insight into cardio-restorative mechanisms supporting scar resolution.

## 1. Introduction

Since the adult heart is incapable of meaningful regeneration, cardiac insult is invariably associated with the deposition of extracellular matrix (ECM) in an adaptive response to repair damaged cardiac muscle. However, prolonged ECM deposition leads to the development of a fibrotic scar that can lead to heart failure and initiate lethal arrhythmias [1,2]. In contrast, the neonatal mammalian heart can generate new muscle after an experimental injury such as apical resection, myocardial infarction, or cryoinjury [3,4,5]. A better understanding of the regenerative capacity of the neonatal heart may facilitate the development of new strategies to support heart repair in the adult.

Cardiomyocytes (CM) exit the cell cycle and become post-mitotic within the first week of life in mice [6,7,8,9]. Although strategies that stimulate CM proliferation improve heart function in experimental ischemic injury models, major barriers to regenerative strategies remain [10,11,12,13,14]. For example, cardiac repair requires poorly understood contributions from diverse cell types. The initial response to apical resection injury consists of thrombus formation, epicardial activation, and cardiac inflammation [15]. The inflammatory response, and the function of macrophages, in particular, is essential for cardiac repair [16]. The role of macrophages is complicated, however, by their direct and indirect contributions to scar formation [16,17]. Cardiac fibroblasts also play a major role in cardiac repair by depositing an ECM-rich scar that ultimately resolves, restoring normal heart muscle structure and function [5,18,19]. While generally considered pathological in heart disease, recent reports document the role of neonatal ECM in supporting cardiomyocyte repopulation and re-vascularization of injured tissue [20,21,22].

Single cell RNA-sequencing and candidate approaches have improved our understanding of heart development, pathologic cardiac remodeling and the contributions of various cells to regenerative heart repair [23,24,25]. However, these studies do not provide spatio-temporal information that describe regional changes in phenotype in an unbiased manner. To overcome this limitation, RNA-sequencing of microdissected heart tissue or genetically labeled cardiac cells has better characterized the developing and remodeling heart [26,27]. More recently, histological sectioning combined with sequencing of all RNAs within a particular section, called Tomo-seq, has defined regional contributions to pathologic fibrosis and cardiac regeneration [28,29]. Through these studies, the border zone (BZ), defined as the region between injured and healthy myocardium, was described as a dynamic tissue with potential importance to cardiac repair [11,27]. The BZ harbors relatively immature cardiomyocytes that undergo metabolic reprogramming during regenerative repair and may be a key source of cardiomyocytes that repopulate damaged tissue [11]. However, the spatial resolution of these approaches is very limited. Spatial transcriptomics has emerged as a powerful method to define regional tissue identities via sequencing of all transcripts that are released from a histological tissue section, following mRNA capture on barcoded oligos with positional information [30,31,32,33].

Here, we used bulk RNA-sequencing of genetically labeled cardiac fibroblasts, combined with spatial transcriptomics of the regenerating neonatal mouse heart, to interrogate phenotypic changes correlating with scar resolution and cardiac repair. Importantly, spatial transcriptomics attained near single cell resolution, identifying the layer of epicardial cells that reside on the surface of the heart. Unbiased transcriptional profiling defined programs of atrial and ventricular chamber maturation, which include unique, spatially distinct gene expression profiles within the compact and trabecular myocardium. We also defined the phenotypic trajectory of the resolving scar, which is characterized by spatially and temporally restricted programs of inflammation, epicardium expansion and ECM production, metabolic reprogramming, scar extrusion, and cardiomyocyte restoration. Coupled with bulk RNA-sequencing of cardiac fibroblasts, we find the regenerative window is biased towards a proliferative phenotype that shifts to a secretory phenotype as the heart matures, even without cardiac insult. Importantly, the resolving scar includes a transcriptionally distinct border zone that exhibits a fetal/immature CM phenotype and that expresses candidate cardio-restorative factors such as *Sprr1a*. Through the unbiased characterization of spatially and regionally distinct transcriptional programs during neonatal cardiac regeneration, this study provides insight into mechanisms supporting scar resolution that may advance novel therapeutic approaches for cardiac repair.

## 2. Materials and Methods

### 2.1. Mice

All experiments involving animals were approved by the University Committee on Animal Resources at the University of Rochester. The C57BL/6J mice were purchased from Jackson Laboratories (stock number 000664). Mice were bred such that a TCF21^MerCreMer/+^ sire was crossed with a Rosa^mTmG/mTmG^ dam to obtain TCF21^MerCreMer/+^; Rosa^mTmG/+^ progeny. Mice were considered postnatal (P) day 0 on the day of birth (P0). For inducible TCF21-lineage labeling, 25 mg/mL 4-Hydroxy tamoxifen (Sigma-Aldrich, St. Louis, MO, USA, H6278) was resuspended in sunflower seed oil, sonicated and administered to 1 day old pups (P1) via subcutaneous injection with a one-time dose of 12.5 mg/kg, in a 1 μL volume using a Hamilton syringe [34,35].

### 2.2. Apical Resection Surgery

All pups in a litter were exposed to apical resection or sham treatment. Prior to surgery, the mother was separated into a clean cage until all surviving pups recovered. Pups were administered slow-release buprenorphine intraperitoneally using a Hamilton syringe, as per the University of Rochester analgesic protocols, with 1 µL for P2 (2 day old) pups and 4 µL for P10 (10 day old) pups, from a stock of 0.04 mg/mL. Apical resections and corresponding sham surgeries were performed as described in the literature [3]. Briefly, pups were anesthetized by hypothermia. A pup was placed within a glove and the glove was placed underneath ice. Appropriate anesthesia was assessed by mouse response, by gently applying pressure to a hind leg. Mice were immobilized to reveal their chest cavity on an ice pack wrapped with surgical drapes and were provided supplemental oxygen. Lateral thoracotomy was performed at the 4th space to expose the heart. The apex was resected using fine-tipped iridectomy scissors. Upon injury, a blood clot formed and prevented exsanguination. For sham surgeries, the heart was exposed through the 4th intercostal space and replaced. The incision was closed with a 7-0 non-absorbable silk suture and the skin was closed using an adhesive. Pups were rubbed with bedding before being placed back into nest. All surviving and recovered pups were returned to the nest before mom was reintroduced. Most P2 pups survived surgery (approximately 90%) while many P10 pups did not (nearly a 50% mortality rate).

### 2.3. Histology and Immunohistochemistry

Upon euthanasia, hearts were extracted and washed with PBS to remove excess blood. Gross images of the heart were acquired on the Zeiss Stereo Discover V12 microscope (Zeiss, Jena, Germany).

Hearts were fixed in 10% Neutral Buffered Formalin (Thermo Fisher Scientific, Waltham, MA, USA) for 24 to 48 h. 5 µm paraffin sections were cut at 5 levels, spaced 100 µm apart. Heart sections were deparaffinized using a Leica Autostainer through a series of xylene, 3 min incubations in 100% ethanol (EtOH, 3×), 95% EtOH (1×), and then were placed in distilled water. Antigen retrieval was performed in pH 6 Dako Target Antigen Retrieval Buffer (Agilent Technologies, Santa Clara, CA, USA) at a high pressure for 15 min. Endogenous fluorescence was quenched in 3% H_2_O_2_ in 150 mM NaCl/100 mM Tris pH 7.5 (TN buffer). Slides were blocked in 0.5% Blocking Reagent (Perkin Elmer, Waltham, MA, USA) in TN buffer. Slides with primary antibodies in blocking solution were incubated overnight (12 to 15 h) at 4 °C. After 10 min washes with TN (3×), secondary antibodies in blocking solution were applied for two hours at room temperature. For signal amplification, Biotin or HRP was applied for two hours during the secondary step, followed by 2 h of incubation with streptavidin in blocking solution or a 15 min incubation with tyramide in TSA Amplification Dilutant (Akoya Biosciences). Following the final incubation, slides were washed for 10 min with water (2×) prior to incubation with 0.5 µL/mL of DAPI (4′, 6-Diamidino-2-Phenylindole, Dihydrochloride, Thermo Fisher Scientific) for 15 min to stain the nuclei. Slides were mounted with Vectashield Anti-face Mounting Media (Vector Labs) and imaged on the Olympus Confocal Microscope IX81 (Olympus Corporation). Antibodies and concentrations used are listed in Table 1.

### 2.4. Bulk RNA Sequencing

#### 2.4.1. Flow Cytometry

In preparation for flow cytometry, each heart was dissected to remove the atria and valve tissue. The apical half of the ventricles was subsequently minced in 1% Bovine Serum Albumin (BSA, Sigma Aldrich, St. Louis, MO, USA) and 0.1% glucose (Fisher Scientific) in Hank’s Balanced Salt Solution without calcium nor magnesium (HBSS, Fisher Scientific). Tissue fragments and solution were collected into a conical tube with 2% Fetal Bovine Serum (GeminiBio, Sacramento, CA, USA) in PBS. After centrifugation and resuspension into collagenase/dispase solution (Sigma Aldrich), samples were shaken at 37 °C for 20 (P2–P5 hearts) to 30 (P10–P13 hearts) minutes. Samples were centrifuged and resuspended in 2% FBS in PBS prior to filtration using a 40 µm filter. Cells were centrifuged and resuspended in 1% BSA/0.1% glucose/HBSS solution for flow cytometry. Endogenous GFP and tdTomato fluorescence were utilized to sort the cells, in which GFP labeled the TCF21-lineage and tdTomato labeled all other cells. BV421 Rat anti-mouse CD140a was used to label the PDGFR-alpha positive cells (BD Biosciences BDB562774 at a 1/100 dilution). DAPI (4′,6-Diamidino-2-Phenylindole, Dihydrochloride) at 1:10,000 was used to label dead cells (Fisher). Cells were sorted using an 18-color BD fluorescence activated cell sorting (FACS) Aria II (BD biosciences).

#### 2.4.2. Low-Input Library Prep

Total RNA was isolated using TRIzol Reagent (Thermo Fisher, Carlsbad, CA, USA) per the manufacturer’s recommendations. RNA concentration was determined with the NanoDrop 1000 spectrophotometer (NanoDrop, Wilmington, DE, USA) and RNA quality was assessed with the Agilent Bioanalyzer 2100 (Agilent, Santa Clara, CA, USA). An amount of 1 ng of total RNA was pre-amplified with the SMARTer Ultra Low Input kit v4 (Clontech, Mountain View, CA, USA) per the manufacturer’s recommendations. The quantity and quality of the subsequent cDNA was determined using the Qubit Fluorometer (Life Technnologies, Carlsbad, CA, USA) and the Agilent Bioanalyzer 2100 (Agilent, Santa Clara, CA, USA). 150 pg of cDNA was used to generate Illumina compatible sequencing libraries with the NexteraXT library preparation kit (Illumina, San Diego, CA, USA) per the manufacturer’s protocols. The amplified libraries were hybridized to the Illumina flow cell and amplified using the NextSeq550 DNA sequencer (Illumina, San Diego, CA, USA). Single end reads of 75 nt were generated for each sample.

#### 2.4.3. Analysis of RNA Sequencing and Statistical Analysis

Raw reads generated from the Illumina base calls were demultiplexed using bcl2fastq version 2.19.1. Quality filtering and adapter removal were performed using FastP version 0.20.0 with the following parameters: “--length_required 35 --cut_front_window_size 1 --cut_front_mean_quality 13 --cut_front --cut_tail_window_size 1 --cut_tail_mean_quality 13 --cut_tail -y -r”. Processed/cleaned reads were then mapped to the Mus musculus reference genome (GRCm38.p6 + Gencode-M22 Annotation) using STAR_2.7.0f with the following parameters: “—twopass Mode Basic --runMode alignReads --outSAMtype BAM SortedByCoordinate–outSAMstrandField intronMotif --outFilterIntronMotifs RemoveNoncanonical –outReads UnmappedFastx”. Gene-level read quantification was derived using the subread-1.6.4 package (featureCounts) with a GTF annotation file (Gencode M22) and the following parameters: “-s 0 -t exon -g gene_name”. Differential expression analysis was performed using DESeq2-1.22.1, with a *p*-value threshold of 0.05 within R version 3.5.1: https://www.R-project.org/ (last accessed 9 March 2021). A PCA plot was created within R using the pcaExplorer to measure sample expression variance. Heatmaps were generated using the pheatmap package and were given the rLog transformed expression values. Gene ontology analyses were performed using the EnrichR package. Statistical analysis for the bulk sequencing data was performed using R or acquired from GeneOntology (PANTHER). Significance is defined as *p* < 0.05.

### 2.5. Spatial Transcriptomics

#### 2.5.1. Sample Preparation

We isolated hearts from mice at 3, 7, 14, and 21 days after they received an apical resection surgery at P2. For cryosection histology, hearts were oriented directly into a cryomold with Optimal Cutting Temperature media. The cryomold was subsequently placed into a prechilled bath of supercooled isopentane, with care taken not to introduce bubbles or alter the orientation of the heart. After OCT was completely frozen, hearts were transferred onto dry ice and stored at −80 °C until they were sectioned. Based on the results of Masson’s Trichrome stained sections, a single representative heart from each timepoint was used for spatial transcriptomics. One hour prior to sectioning, slides and hearts were equilibrated in the prechilled cryostat at −17 °C. Cryosections (12 µm) were cut and placed carefully onto the specified capture area (containing the barcoded probes) on the 10X Visium Spatial Gene Expression Slide (10X Genomics, Pleasanton, CA, USA). Upon placing all sections appropriately on the slide, the slide was stored at −80 °C for up to a week.

For H&E staining and imaging of the tissue section that would subsequently be used for sequencing, tissue sections were fixed in methanol and were H&E stained, following the Visium Methanol Fixation and H&E Staining guide. Briefly, the slides were incubated for 1 min at 37 °C prior to a 30-min incubation at −20 °C in prechilled methanol. Slides were uniformly covered with 500 µL isopropanol and incubated at room temperature for 1 min. Slides were air dried for up to 10 min before a 7 min incubation at room temperature with 1 mL hematoxylin. Slides were washed by dipping the slide in fresh water 15×). Slides were incubated with 1 mL Bluing Buffer at room temperature for 2 min before dipping the slide in water (5×). Slides were incubated with 1 mL Eosin for 1 min at room temperature before dipping the slide into water (15×). Slides were incubated on a thermocycler (BioRad) for 5 min at 37 °C before imaging on a VS120 Slide Scanner (Olympus, Center Valley, PA, USA). Images were saved for each capture area as a 2000 × 2000 pixel TIFF for downstream analysis.

#### 2.5.2. mRNA Capture and Library Generation

After permeabilization optimization per the Visium protocol, tissue was permeabilized for 18 min and poly-adenylated mRNAs were released and captured by poly-dT oligos within the Visum slide capture area. Reverse transcription was performed to produce full-length cDNA using the Visium Spatial Gene Expression Kit, as per the manufacturer’s recommendation. cDNA was assessed by KAPA qPCR to determine optimal cycle numbers for cDNA amplification. Post-amplification, cDNA was purified using SPRISelect beads (ThermoFisher) and quality was assessed with the Qubit Flourometer (Life Technnologies, Carlsbad, CA, USA) and the Agilent Bioanalyzer 2100 (Agilent, Santa Clara, CA, USA). An amount of 25% of total cDNA yield was used to generate Illumina-compatible sequencing libraries with the Visium Library Construction Kit. Enzymatic fragmentation and size selection was used to optimize the cDNA amplicon size and indexed sequencing libraries were constructed by End Repair, A-tailing, Adaptor Ligation, and PCR. The amplified libraries were hybridized to the Illumina flow cell and sequenced using the NovaSeq 6000 sequencer (Illumina, San Diego, CA, USA) following the sequencing requirements in the Visium Spatial Gene Expression manual of 28 × 10 × 10 × 90.

#### 2.5.3. Statistical Analysis of RNA Sequencing Data

The 10x SpaceRanger software (v1.0) was used to perform demultiplexing and derive the raw count values for each barcoded spot. Loupe Browser was used in validation studies to assign regions from the captures to a particular structure using anatomical segmentation. Using the lasso tool, barcoded spots, corresponding to the identified anatomical regions, were selected on Loupe Browser (10X Genomics). Significantly upregulated and downregulated genes within each region were selected from the gene lists generated by the Loupe Browser. Significance was determined by a value of *p* < 0.05 and a fold change of 2 or greater. Significant genes were subsequently analyzed for biological gene ontology terms using PANTHER.

Seurat (v4.0) was used to analyze and integrate the captures for cross-capture comparisons. We applied cell cycle scoring per Seurat standards after mapping the human genes included within Seurat’s package to the mouse using biomaRt: https://satijalab.org/seurat/articles/cell_cycle_vignette.html (last accessed 9 March 2021). While we labelled spots by their cell cycle, we did not regress out the cell cycle features for the purposes of clustering. Marker genes for cross-capture cell types were determined using FindAllMarkers and used for pathway enrichment within enrichR (v3.0) and clusterProfiler (v3.18.1). Cell type clusters residing in the scar of the apex were subsetted out, renormalized, and used to generate a pseudotime trajectory with a monocle (v2.18.0). Marker genes from the generated pseudotime states were used within enrichR and clusterProfiler for further pathway enrichment. All statistical analysis were performed using R. Significance is defined as *p* < 0.05.

## 3. Results

### 3.1. Acquisition of the Secretory Cardiac Fibroblast Phenotype Corresponds with the Loss of Regenerative Potential

**Figure 1:**
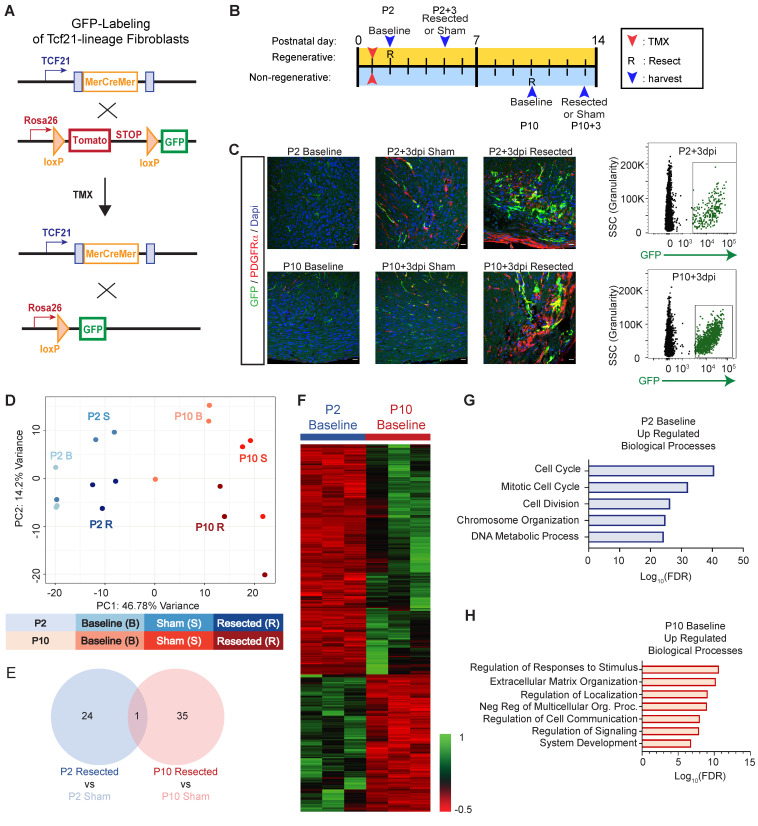
RNA sequencing of resident cardiac fibroblasts reveals a switch to secretory phenotype which corresponds to loss of regenerative capacity. (**A**) Schematic describing the breeding strategy used to generate mice that allowed fluorescent lineage training of resident fibroblasts. (**B**) Schematic describing experimental strategy, including timing of tamoxifen (TMX) injections, apical resection or sham surgeries (R), and fibroblast and tissue isolations. (**C**) Hearts were obtained from postnatal mice at indicated timepoints, and stained with antibodies to visualize GFP (Tcf21+ fibroblast lineage), PDGFR-a (endothelial cells and fibroblasts) and DAPI (nuclei). Representative flow cytometry plots are shown on the right that indicate the population of GFP+ fibroblasts obtained for RNA-sequencing. (**D**) Principal component analysis (PCA), revealing independent gene expression programs based on RNA-sequencing of cardiac fibroblasts of indicated treatment. **(E)** Venn diagram representation of differentially expressed genes in response to apical resection at indicated timepoints. (**F**) Heat map representation of genes that exhibit differential expression between P2 and P10 at baseline. (**G**) Biological processes that are enriched in cardiac fibroblasts at P2, compared to P10. (**H**) Biological processes that are enriched in cardiac fibroblasts at P10 compared to P2.

The primary source of ECM in the heart is resident cardiac fibroblasts, which respond to cardiac insult by generating a fibrotic scar [36,37]. Following cardiac insult in zebrafish, fibroblast activation appears to be transient, and the scar subsequently resolves as the heart regenerates [19,38]. Since immature CM maintains a proliferative potential, the neonatal heart regenerates after an injury that occurs shortly after birth [5]. Cardiac insult prior to CM becoming post-mitotic results in a resolving scar, while an injury after this time produces an irreversible scar. To interrogate the fibroblast response in regenerative cardiac injury, compared to non-regenerative injury, we used the Tcf21^MerCreMer^ mouse line, which expresses the tamoxifen-inducible Cre recombinase in resident cardiac fibroblasts (Figure 1A) [39,40]. To track cardiac fibroblasts during repair from apical resection surgery, Tcf21^MerCreMer/+^; Rosa^mTmG/+^ pups were injected with tamoxifen at P1, and then given an apical resection or sham surgery at P2 (regenerative timepoint) or P10 (non-regenerative timepoint) (Figure 1B). Immunofluorescence imaging revealed an accumulation of GFP+ and PDGFRα+ fibroblasts with postnatal age, and within the cardiac apex following an apical resection injury when compared to the sham controls (Figure 1C). We note that Tcf21-lineage fibroblasts did not completely overlap with PDGFRα+ cells at this timepoint.

To identify the phenotypic changes occurring in response to heart maturation or injury within the regenerative and non-regenerative time points, we next collected GFP-labeled TCF21-lineage fibroblasts via FACS at 3 days post-injury (dpi) or sham surgery, and also from un-manipulated mice at P2 and P10 (Figure 1C). Following bulk RNA sequencing, the principal component analysis (PCA) revealed distinct transcriptional signatures based on postnatal age, and in response to apical resection surgery (Figure 1D). Surprisingly, despite the expansion of TCF21-lineage fibroblasts at the apex of the heart following resection, we only identified 25 differentially expressed genes (DEGs) between sham or resection surgery groups at P2 +3 days (Figure 1E and Table S1). DEGs at P2 resection vs. sham were enriched in pathways associated with phosphatidic and glycerophospholipid biosynthesis (Figure S1A). Likewise, apical resection surgery at P10 significantly altered the expression of only 36 genes, which were associated with the regulatory control of organelle and cellular components (Figure 1E, Figure S1B and Table S2). In contrast, the transcriptome of fibroblasts obtained within the regenerative window (P2 baseline, P2 sham +3 days, and P2 resected +3 days) was significantly different from fibroblasts obtained within the non-regenerative window (P10 baseline, P10 sham +3 days, and P10 resected +3 days), regardless of surgical intervention (Figure 1D). Hierarchical clustering revealed considerable transcriptional differences in fibroblasts obtained from P2 and P10 hearts at baseline (Figure 1F and Table S3). Genes that were upregulated in fibroblasts at P2 when compared to P10 include *Cdk1*, *Ccna2*, *Ccnb1,* and *Ccnb2*, revealing an enrichment in Gene Ontology (GO) terms associated with proliferative pathways, such as the cell cycle, chromosome organization, and cell division (Figure 1G). Genes that are upregulated in fibroblasts at P10 include *Pcolce2*, *Thbd*, and *Cilp*, revealing an enrichment in GO terms related to a secretory phenotype, including extracellular matrix organization, regulation of cell communication, and system development (Figure 1H). Importantly, while bulk RNA-seq identified bias towards a secretory fibroblast gene program in the non-regenerative period that may predispose the heart to scar formation, this study did not reveal major changes in fibroblasts following injury. It is possible that bulk RNA-sequencing lacks the sensitivity to detect changes that occur in a limited number of fibroblasts that respond to injury, especially in the context of the broad phenotypic changes we observed during postnatal maturation.

### 3.2. Interrogating Postnatal Heart Maturation and Regeneration Using Spatial Transcriptomics

#### 3.2.1. Verifying Efficacy of Spatial Transcriptomics

**Figure 2:**
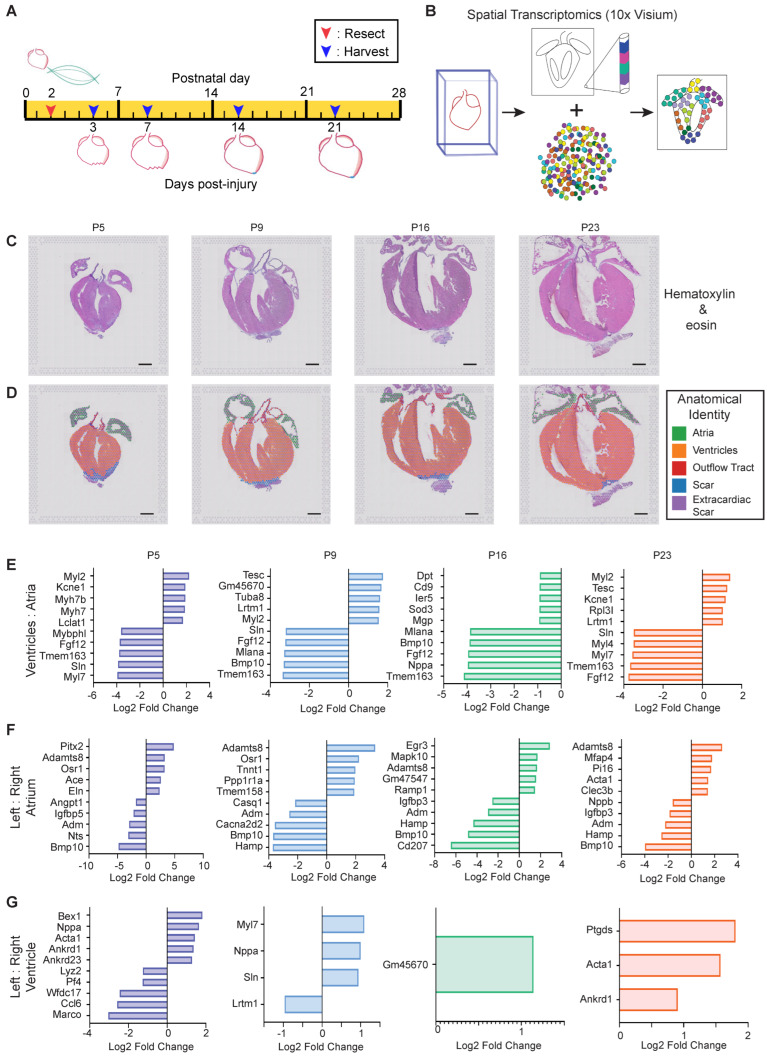
*Spatially and temporally resolved transcriptional map of the regenerating neonatal mouse heart.* (**A**) Schematic representation of the experimental timeline of apical resection (R) and tissue harvest for spatial transcriptomics. (**B**) 10X Visium spatial transcriptomics experimental strategy. A single representative heart was evaluated by spatial transcriptomics for each timepoint on a single slide. (**C**) Hematoxylin & Eosin stained sections used for spatial transcriptomics. (**D**) Loupe Browser was used to assign regional posts an anatomical identity, indicated by color. (**E**–**G**) Rank ordering of differentially expressed genes at timepoints and comparison indicated in the graph reveals the most-enriched genes that define the (**E**) ventricles versus atria; (**F**) left versus right atrium; or (**G**) left versus right ventricle. Scale bar = 1 mm.

Functionally meaningful transcriptional changes that occur only within a small region of tissue may be overwhelmed by transcripts from the rest of the tissue when using bulk RNA-sequencing approaches. To address spatially restricted transcriptional programs in postanal heart maturation that may contribute to cardiac regeneration, we utilized spatial transcriptomics, a technique to integrate gene expression data and location within a histological tissue section [33]. The neonatal heart was resected at P2 and collected at 3, 7, 14, and 21 dpi for spatial transcriptomics (Figure 2A). Freshly cryopreserved hearts were sectioned and placed on one of four capture areas on a 10X Visium slide, which each contains ~5000 spots with unique spatially barcoded capture oligo probes. H&E staining and high-resolution imaging was performed prior to the permeabilization of tissue and mRNA capture, to allow for the cross-referencing of transcriptomics data to histological features. Following permeabilization, mRNAs released from the tissue section were captured by polyT tails on the capture probe for library preparation and RNA-sequencing. Each sequencing read was then be mapped to a location in the H&E image corresponding to the spatial barcode (Figure 2B). Following RNA-sequencing, we first validated that spatial transcriptomics recapitulates the pattern of genes known to exhibit restricted expression within the heart. Using Loupe Browser, we manually assigned a capture spot to a cardiac structure, including atria, ventricles, outflow tract, scar, and extracardiac tissue, using anatomical segmentation (Figure 2C,D). Extracardiac tissue was specified as any tissue beyond the natural apex of the heart and scar tissue was defined as fibrotic tissue within the heart, based on H&E staining.

Rank ordering of genes enriched in the ventricles when compared to the atria (Figure S2A) revealed the expected enrichment in *Myl2* (myosin light chain 2), while the atria robustly expressed *Nppa*, *Fgf12,* and *Sln* (Figure 2E) [41,42,43]. Of note, we observed an initial enrichment of *Myh7* and *Myh7b* in the ventricles at P5, which subsided over the course of postnatal heart maturation, reflecting myosin isoform switching (Figure 2E). We also investigated the differences between the left and right chambers as defined anatomically (Figure S2B,C). When compared to the left atrium, the right atrium highly expressed genes associated with vascular development (*Adm* and *Hamp*) and cardiac growth (*Bmp10*) (Figure 2F) [44,45,46,47]. The left atrium was enriched in *Adamts8* at all timepoints analyzed [48]; at P5, the left atrium was especially enriched in *Pitx2*, which was previously shown to confer left atrial identity (Figure 2F) [49]. In contrast to the atria, differences between the left and right ventricles as defined by spatial transcriptomics were less remarkable (Figure 2G). As expected, we observed an enrichment of *Nppa* transcripts in the early postnatal left ventricle; however, by P16 there were few DEGs remaining. These results are generally consistent with single cell RNA-sequencing of microdissected chambers in the embryo [23].

#### 3.2.2. Cellular Resolution of Unbiased Spatial Transcriptomics

**Figure 3:**
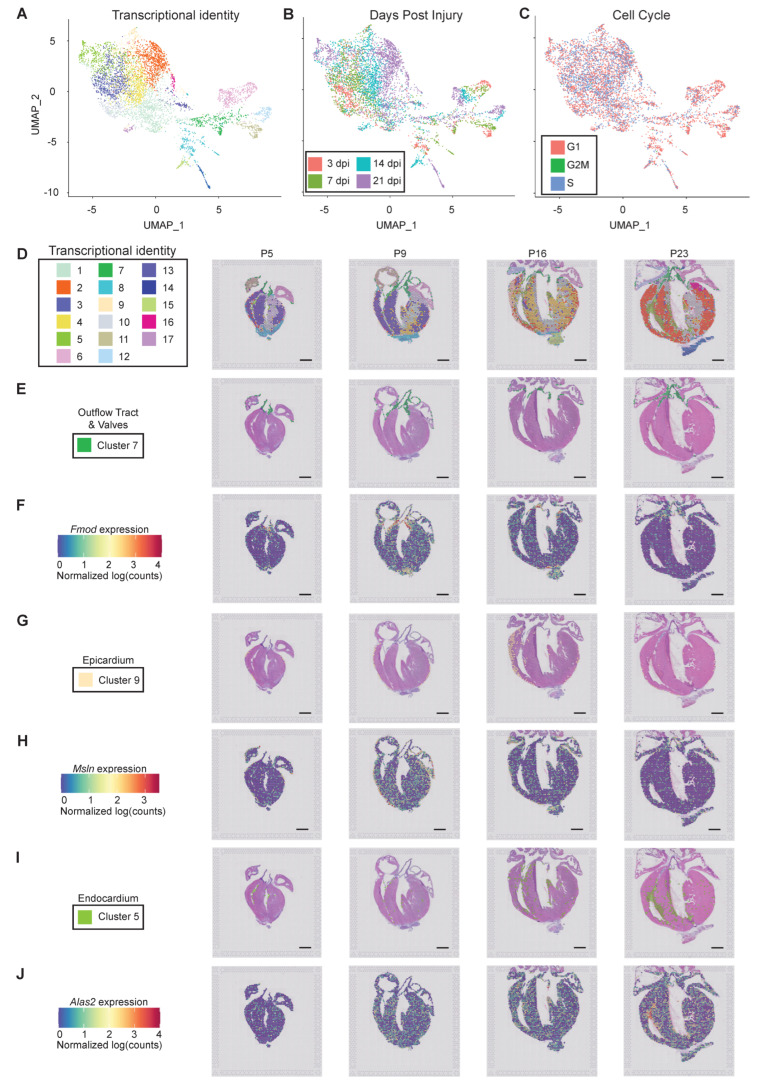
Spatial transcriptomics reveals spatially restricted gene programs in postnatal mouse heart regeneration at near single-cell resolution. (**A**) Uniform manifold approximation and projection (UMAP) was generated using Seurat to visualize barcoded spots with similar transcriptional identity based on RNA-sequencing. (**B,C**) Barcoded spots within UMAP were labeled based on (**B**) days postinjury (dpi); and (**C**) phase of the cell cycle. (**D**) Barcoded spots representing 17 transcriptionally distinct clusters were mapped to their origin within a tissue section for the unbiased identification of anatomical structures with unique gene expression programs. (**E**) Localization of cluster 7 spots reveals localization to the outflow tract and valves. (**F**) Spatially resolved Fmod expression reveals enrichment in heart valves. (**G**) Localization of cluster 9 spots reveals localization to the epicardium. Note the single layer of spots that labels the epicardium on the heart’s surface, and the epicardial expansion observed at 14 dpi. (**H**) Spatially resolved Msln expression reveals enrichment in heart valves. **(I)** Localization of cluster 5 spots reveals localization to the endocardium. (**J**) Spatially resolved Alas2 expression reveals enrichment in endocardium, especially as heart maturation proceeds. Scale bar = 1mm.

Since we verified spatial transcriptomics as a robust method to detect regional cardiac gene programs using a candidate approach, we next performed an unbiased transcriptomics analysis using Seurat. Uniform manifold approximation and projection (UMAP) analysis defined 17 unique transcriptional signatures (Figure 3A); some clusters appeared to be primarily composed of spots from a single postnatal timepoint. which displayed a strong correlation with developmental age but not with the cell cycle (Figure 3B,C). Integrating transcriptional information from the capture spots to spatial location revealed broad clustering within morphological structures such as the upper chamber, lower chamber, and scar, as well as detailed sub-clustering within chambers (Figure 3A,D). For example, transcriptional annotation defined the outflow tract and semiluminar valves (cluster seven), which showed highly specific localization within the tissue section (Figure 3D,E). Cluster seven is defined by 299 DEGs, including genes correlated with ECM remodeling (*Adamts2, Cilp2, Fbln1, Fmod Ltbp3,* and *Timp3*) [50,51,52,53,54,55]. The most enriched genes within cluster seven include *Fmod* and *Fbln5*, which mark the valves and ascending aorta, respectively (Figure 3F and Figure S3A). *Cilp2* was also highly enriched in valve tissue, in addition to the atria (Figure S3B). Upregulated gene expression programs in cluster seven include ECM organization, neutrophil and platelet degranulation (Figure S3C).

Importantly, the epicardium (cluster nine) and endocardium (cluster five) were also defined transcriptionally. Thus, spatial transcriptomics resolves a tissue comprised of a single layer of mesothelial or endothelial cells, respectively (Figure 3G–J). The single layer of epicardial cells was partially defined by the differential expression of 72 genes, including *Msln* (Figure 3F). Of note, we observed a thickening of the epicardial layer at P16, corresponding to 14 days post apical resection injury (Figure 3G). The endocardium was most apparent at P16 and P23, and was defined by the expression of 115 genes, including *Alas2*, which was unique to this tissue type (Figure 3J). Together, these data reveal the remarkable resolution of unbiased spatial transcriptomics, which is capable of defining a tissue type that is composed of a single cell layer.

#### 3.2.3. Atrial Chamber Maturation

**Figure 4:**
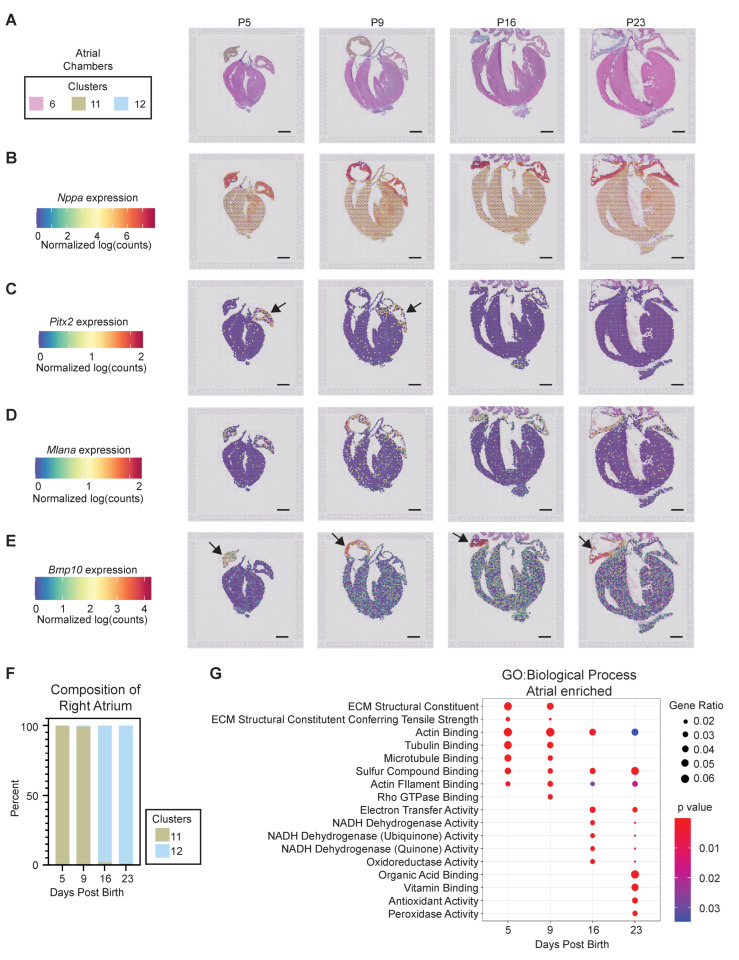
*Spatial transcriptomics defines postnatal program of atrial chamber maturation.* (**A**) Barcoded spots representing the 3 transcriptionally distinct atrial clusters were mapped to their origin. (**B–E**) Spatially resolved expression of atrial enriched genes identified by spatial transcriptomics reveals (B) *Nppa* enrichment in both atria at all timepoints, and trabecular myocardium at P5 and P9; (**C**) *Pitx2* enrichment in the left atrium, especially at P5 and P9; (**D**) *Mlana* expression enriched in the right atria, especially after P5; and (**E**) *Bmp10* enrichment in the right atrium at all timepoints. Arrows indicate left (**C**) or right (**E**) atrium. (**F**) Right atrium maturation program is revealed consisting of a shift from cluster 11 identity at P5 and P9 to cluster 12 identity at P16 and P23. **(G**) Biological processes were interrogated as a function of postnatal time, revealing a shift from ECM and actin/microtubule processes to oxidative metabolism as the atria mature. Scale bar = 1 mm.

We next investigated tissue-restricted gene profiles corresponding to postnatal chamber maturation. Unbiased clustering defined three primary atrial identities, (clusters 6, 11, and 12), all expressing high levels of *Sln* and *Nppa* (Figure 4A,B and Figure S4A). The left atrium was composed entirely of cluster six posts throughout all postnatal timepoints (Figure 4A), and is defined by 121 DEGs with a fold change of two or greater when compared to all other clusters in the computational analysis, including the notable expression of *Tbx5* and, *Sfrp1*, and *Pitx2* (Figure 4C, Figure S4B and Table S4) [49,56,57]. In contrast, the right atrium displays a striking enrichment in the expression of *Mlana* and *Bmp10* when compared to the left atrium, at all times (Figure 4D,E). Of note, the right atrium also exhibits a phenotypic shift between early and late postnatal stages. The right atrium is characterized by cluster 11 at P5 and P9, which shifts to an abundance of cluster 12 spots at P16 and P23, revealing that atrial identity is significantly influenced by postnatal age (Figure 4A,F). GO terms analysis defined a progression from extracellular matrix organization and tubulin/microtubule binding at P5 and P9, to cellular respiration at P16 and P23 (Figure 4G). At early postnatal timepoints, the right atrium is enriched in genes, including *Adm, Dbh, Hey1, Ryr3,* and *Smarcd3,* which have been correlated with the right atrium phenotype [57]. Additional cluster 11-enriched genes are associated with gap junctions (*Gja5*), angiogenesis, and inflammation (*Mdk*), as well as cytoskeletal components (*Tuba4a* and *Tuba8*) (Figure S4C and Table S4) [58,59,60]. Genes enriched in the mature right atrium cluster 12 include *Hamp*, *Cd207*, *Igfbp3*, and *Vwf* (Figure S4D and Table S4). Differentially expressed genes in cluster 12 are also associated with vesicular trafficking (*Doc2g*) and intracellular signaling (*Gng2*) [61,62].

#### 3.2.4. Ventricular Chamber Maturation

**Figure 5:**
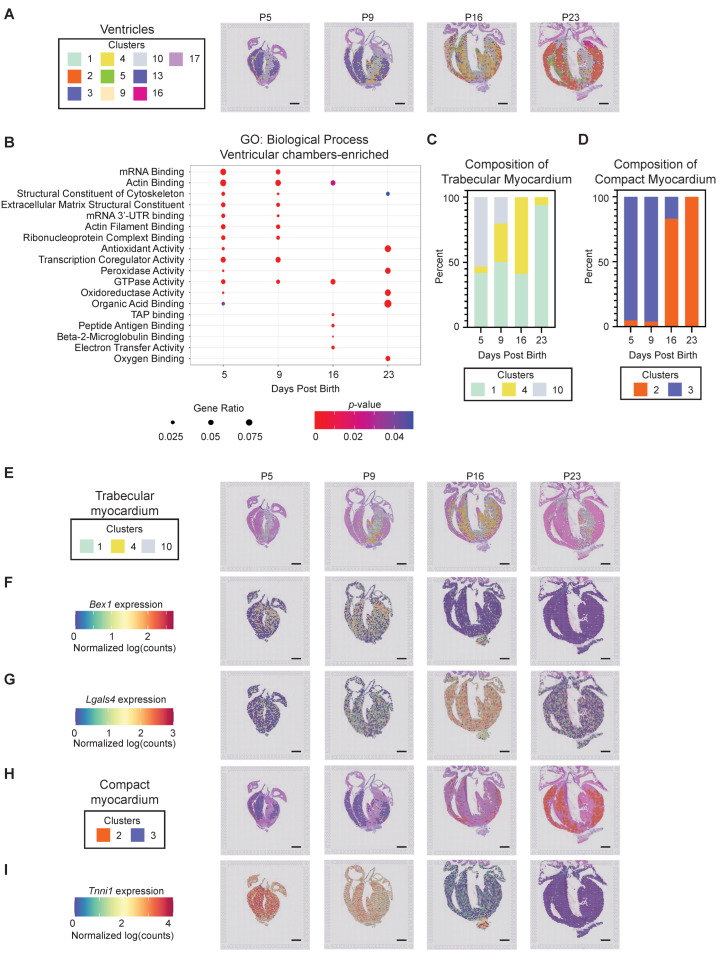
*Spatially resolved transcriptional programs define trabecular and compact ventricular myocardium maturation.* (**A**) Barcoded spots, representing the 10 transcriptionally distinct ventricular clusters, were mapped to their origin. (**B**) Biological processes were interrogated as a function of postnatal time, revealing a shift from ECM, actin binding, and cytoskeleton processes to oxidative metabolism as the ventricles mature. (**C**) Trabecular myocardium maturation program is revealed, consisting of a shift from 1 and 10 at P5, through an intermediate identity with a high percentage of cluster 4, to being dominated by cluster 1 at P23. (**D**) Compact myocardium maturation program is revealed, consisting of a shift from cluster 3 identity at P5 and P9 to cluster 2 identity at P16 and P23. (**E**) Barcoded spots with trabecular myocardium identity were mapped back to an anatomical origin. (**F**,**G**) Spatially resolved expression of trabecular enriched genes identified by spatial transcriptomics reveals (**F**) *Bex1* expression within trabecular myocardium, especially at P5 and P9; and (**G**) *Lgals4* expression, particularly at the intermediate P16 timepoint. (**H**) Barcoded spots with compact myocardium identity were mapped back to an anatomical origin. (**I**) Spatially resolved *Tnni1* expression reveals a decrease within compact and trabecular myocardium as the heart matures. Scale bar = 1 mm.

Spatial transcriptomics resolves ventricular tissue into 10 subpopulations that include the epicardium, endocardium, trabecular myocardium, compact myocardium, and ventricular septum (Figure 5A). GO term analysis, carried out on all ventricular tissue types as a function of postnatal age, revealed a general transition from extracellular matrix and cytoskeletal structural components to metabolism and oxidoreductase activity (Figure 5B). We observed the expected switch from *Myh7* to *Myh6* as postnatal heart maturation proceeded (Figure S5A,B). Interestingly, unbiased clustering also defined unique maturation signatures for the trabecular and compact myocardium. The trabecular myocardium is characterized by three transcriptionally distinct phases defined by clusters 1, 4 and 10 (Figure 5C). Cluster 10 is most abundant at P5, and gradually regresses, while cluster four primarily defines trabecular myocardium at transitional postnatal ages (P9 and P16). Cluster one is present at all ages, and becomes the predominant identity within P23 trabecular myocardium. Compact myocardium is defined by two independent clusters, initially represented primarily by cluster three, and becoming entirely composed of cluster two identity posts by P23 (Figure 5D). Visualization of the trabecular myocardium identity revealed an abundance of genes, such as *Bex1,* at early postnatal stages (Figure 5E,F) [63]. Transitional trabecular myocardium is especially enriched in *Lgals4* (Figure 5E,G), while the mature trabecular myocardium (cluster one) is represented by only eight DEGs; only one (*Nppa*) of these exhibited a fold change greater than two (Table S4). *Nppa* was downregulated at P23, as expected for a component of the fetal ventricular gene program. DEGs defining the later postnatal age compact myocardium in cluster two had functions related to cell motility (*Dynll1* and *Actg1*) and collagen deposition (*Col4a1* and *Col5a1*) [64,65]. In addition to *Myh7*, spatial transcriptomics also detected the significant suppression of *Tnni1* expression in the compact myocardium as the heart matured (Figure 5H,I). These data provide a novel framework to explore the transcriptional processes that impede the cell cycle and drive CM maturation in the postnatal heart, which may also be informative for regenerative medicine strategies.

### 3.3. Interrogating Regenerative Scar Resolution Using Spatial Transcriptomics

**Figure 6:**
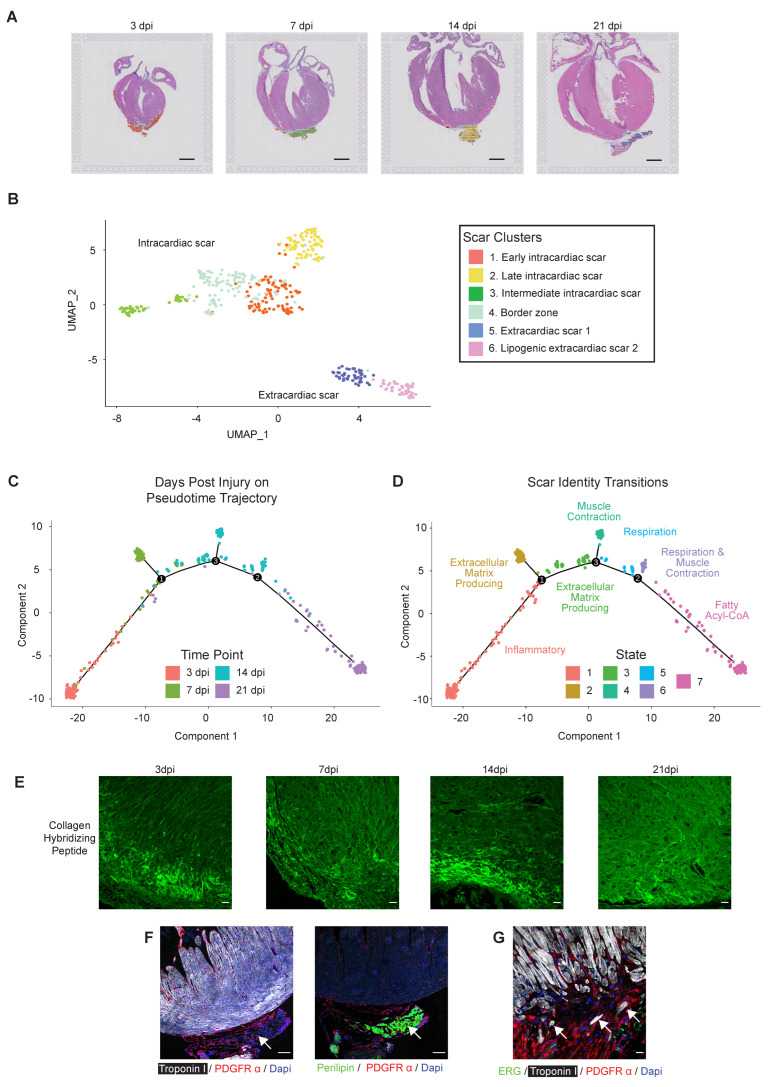
Spatial transcriptomics reveals the phenotypic trajectory of the regenerative scar, consisting of bi-phasic collagen deposition and culminating in scar extrusion. (**A**) Barcoded spots with scar identity were mapped back to an anatomical origin. (**B**) Uniform manifold approximation and projection (UMAP) was generated using Seurat to visualize 6 regional identities within the regenerative scar. (**C**) The developmental trajectory of scar-associated spots was integrated with days post-injury (dpi). (**D**) Pseudotime trajectory was integrated with GO term enrichments to establish phenotypic timeline. (**E**) Representative heart sections obtained at the indicated time after apical resection surgery were stained with collagen hybridizing peptide (CHP) to visualize nascent and remodeling collagen at the cardiac apex. This trend was observed in n = 3/5 independent samples (3 dpi) and n = 4/4 independent samples (14 dpi). (**F**,**G**) Representative heart sections obtained at 21 dpi were stained with antibodies directed against Troponin I (cardiomyocytes, white), PDGFR-a (fibroblasts, red) and perilipin (adipocyte, green in left image) and ERG (endothelial cells, green right image). DAPI stains nuclei (blue). Note the extracardiac fibro-lipogenic scar emerging from the apex (left), that often harbors individual cardiomyocytes (white arrows, right image). Scale bar = 1 mm (**A**) and 100 mm (**E**–**G**).

We next analyzed the spatial transcriptomics data to investigate the healing scar using Seurat, which revealed six scar subtypes composed of four clusters within the intracardiac scar, and two clusters within the extracardiac scar (Figure 6A,B). We also defined independent transcriptional signatures reflecting an early, intermediate, and late scar and a distinct border zone (BZ) region between the cardiac scar and healthy myocardium. We utilized Monocle to establish the developmental trajectory of the scar-associated spots, correlating postnatal age with phenotypic state (Figure S6A,B). Importantly, the pseudotime trajectory displayed a striking positive correlation with days post-injury (Figure 6C). The transcriptional identity of the intracardiac scar-associated states revealed an initial inflammatory response at 3 dpi, represented by an enrichment in Gene Ontology terms such as neutrophil chemotaxis and neutrophil migration in State one (Figure 6D and Figure S7A–D). The intracardiac scar later transitions to a fibrotic response at 7 and 14 dpi, represented by an enrichment in Gene Ontology terms such as ECM assembly, elastic fiber assembly, and ECM organization in States two and three (Figure 6D and Figure S7B–D). Finally, the intracardiac scar terminates with a contractile phenotype by 14 dpi, represented by enrichment of Gene Ontology terms such as muscle contraction, actin–myosin filament sliding, and cardiac muscle tissue morphogenesis in States four to six (Figure 6D and Figure S7E,F). Surprisingly, the end of the pseudotime was nearly entirely composed of 21 dpi spots displaying a lipogenic profile and residing outside of the confines of the heart (Figure 6C,D and Figure S7G).

#### 3.3.1. Bi-Phasic Deposition of ECM

To investigate the dynamics of extracellular matrix deposition and resolution in the regenerating heart, we next assessed the progression of scar resolution and remodeling, based on the distribution of denatured collagen using collagen-hybridizing peptide (CHP) at 3, 7, 14, and 21 days after a P2 apical resection. We observed strong and diffuse CHP staining at 3 dpi, which had slightly resolved by 7 dpi (Figure 6E). We observed a second peak of CHP staining at 14 dpi; unlike at 3 and 7 dpi, denatured collagen at 14 dpi appeared more compact and organized at the cardiac apex. Taken together, CHP staining revealed a biphasic trend of collagen deposition/remodeling, with an initial increase at 3 dpi followed by a more robust increase at 14 dpi. These timepoints are consistent with an initial burst of collagen secretion within the inflammatory phase, perhaps by macrophages as previously reported [17], followed by a second burst of deposition within the secretory phase, which was defined by our bulk RNA-seq of cardiac fibroblasts. The decreased CHP staining intensity at 7 and 21 dpi may be due to combined action of collagen fiber maturation and their decreased deposition at transitional timepoints.

#### 3.3.2. Extracardiac Cardiomyocytes and Lipogenic Tissue

While scar-associated genes were diminished within the boundary of the heart by 21 dpi, a region of extracardiac tissue consistently persisted adjacent to the apex. Genes enriched in this extracardiac tissue (State seven) defined pathways associated with adipogenesis and fatty acyl CoA signaling. Many fibrosis-associated pathways were downregulated, including ECM and collagen fibril organization (Figure S7G). To further evaluate the cellular composition of the extracardiac tissue, resected hearts were stained for perilipin, a marker for lipid droplets. Among hearts resected at P2 and collected 21 dpi, perilipin positive areas were consistently present in the extracardiac tissue beyond the apical boundary of the regenerated heart (Figure 6F). The perilipin-positive region was tightly associated with PDGFRα positive fibroblasts; however, PDGFRα+ fibroblasts were generally not perilipin positive. We also observed isolated Troponin I + CM within the extracardiac tissue at the apex of the heart (Figure 6G). It is possible that the extracardiac tissue represents lipogenic pericardial tissue or adhesions to the body wall; however, extracardiac tissue with a lipogenic profile was not observed earlier in the healing response. It is interesting to speculate that this extracardiac tissue represents the remnants of the healing scar, composed of fibroblasts and CM being extruded through the damaged epicardium and acquiring a lipogenic phenotype.

#### 3.3.3. Defining the Regenerative Border Zone

**Figure 7:**
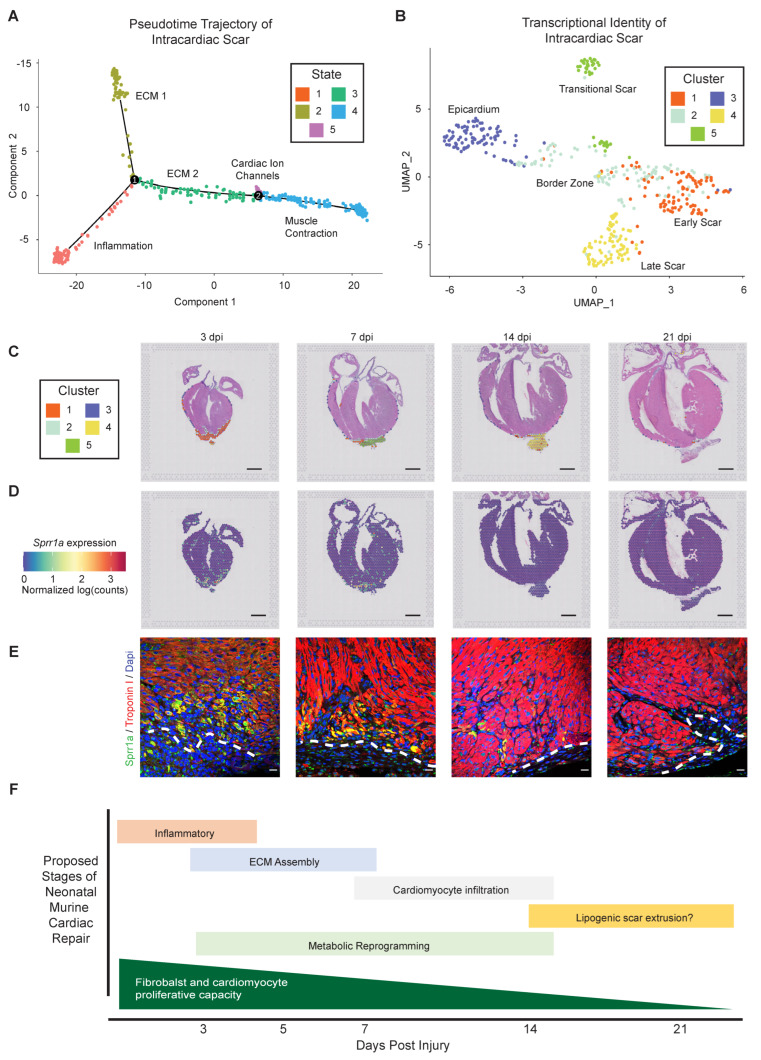
*Spatial transcriptomics reveals the phenotypic trajectory of the intracardiac resolving intracardiac scar and border zone.* (**A**) Pseudotime developmental trajectory was integrated with biological process enrichment to identify the regional identity of the 5 intracardiac scar states. (**B**) Uniform manifold approximation and projection reveals 5 distinct transcriptional clusters that define the temporal progression of the intracardiac scar, the border zone, and the epicardium. (**C**) Barcoded spots were mapped back to an anatomical origin to spatially and temporally resolve the intracardiac scar identity, and location of the border zone as a narrow band of tissue between the injury and healthy myocardium. (**D**) Spatially resolved *Sprr1a* expression reveals an enrichment within the border zone specifically at 3 and 7 dpi. (**E**) Representative heart sections obtained at indicated post-injury timepoints were stained with antibodies directed against Troponin I (cardiomyocytes, red) and Sprr1a (green). DAPI labels nuclei, and the dashed line indicates transition from injury to healthy myocardium, defined as the border zone. Note the enrichment of Sprr1a in border zone cardiomyocytes at 3 dpi and 7 dpi, which is absent in remote myocardium. (**F**) Schematic of the proposed stages of neonatal mouse heart regeneration, based on bulk RNA-sequencing of isolated fibroblasts and spatial transcriptomics at key regenerative timepoints. Scale bar = 1 mm (**C**,**D**) and 100 mm (**E**).

We next removed the extracardiac cluster seven and reanalyzed just the intracardiac scar-associated clusters using Monocle to gain a more specific representation of scar resolution, again revealing a striking correlation with developmental state and time post-injury (Figure S8A–C). Early pseudotime was nearly entirely composed of spots from 3 dpi, becoming biased towards 14 and 21 dpi posts at the end of the pseudotime. The majority of the intracardiac scar followed a trajectory from State one through State three, terminating at States four and five, with an independent terminal in State two (Figure 7A). Pseudotime State one is defined by an enrichment in genes associated with chemokine signaling, platelet degranulation, and neutrophil chemotaxis, as well as the depletion of genes associated with pathways such as mitochondrial and cell respiration processes (Figure S9A). The resolving scar can follow a trajectory terminating in State two, which is enriched in 7 dpi spots that are defined by ECM biological processes (Figure 7A and Figure S9B). Alternatively, the scar transitions through State three, which includes contributions from all the post-injury time points assessed, and is also enriched in ECM-associated pathways, indicating a transitional fibrotic state (Figure S9C). Following transitional State three, the scar phenotype terminates in States four and five, which are characterized by the upregulation of cardiac ion channel activity, muscle filament sliding, and heart contraction pathways, consistent with repopulation of the scar with functional cardiac muscle at 14–21 dpi (Figure S9D,E). Thus, the intracardiac scar follows two paths from the initial inflammatory state: (1) acquisition of a terminal ECM-rich fibrotic state at 7 dpi; or (2) transition through an ECM-rich state prior to acquiring a terminal cardiac muscle identity. It is currently not clear what the ultimate fate of the State two scar trajectory is; fibroblasts may remain in the heart and acquire a senescent matrifibrocyte fate [66], undergo apoptosis and become resolved, or be extruded from the heart to acquire a lipogenic fate as suggested above.

We next utilized Seurat to further spatially and temporally interrogate scar identity, defining five intracardiac scar clusters as early, transitional, and late scar, as well as BZ and epicardium (Figure 7B). Evaluated spatially, the early healing scar is primarily composed of cluster one (Figure 7C), and is defined by pathways associated with inflammatory signaling. Cluster three is localized to the epicardium, a single layer of mesothelium on the surface of the heart that gives rise to many supporting cells, including fibroblasts. While only the apex of the epicardium was damaged by the resection surgery, our overall analysis revealed significant epicardial thickening at 14 dpi, consistent with epicardial reactivation and thickening that has been noted after adult myocardial injury [67,68]. Clusters four and five correspond to intermediate and late scar tissue associated with ECM-related pathways that transition to muscle contraction; thus, we can use spatial transcriptomics to spatially resolve the repopulation intracardiac scar with cardiac muscle at 7–14 dpi, which is nearly completely regenerated by 21 dpi (Figure 7A–C).

Of note, cluster two is represented by a narrow band of spatially resolved spots localized between the scar and the healthy myocardium at all time points. This BZ is an area of active regeneration, involving CM proliferation and expansion into the scar as it resolves; thus, we evaluated this region in more detail. The BZ consists of 15 DEGs, including three unique genes: *Ankrd1*, *Ccl21*, and *Sprr1a*. *Ankrd1* is a marker of immature CM observed during embryonic development, and also of cardiac diseases [27,69,70,71], while *Sprr1a* is correlated with cardioprotection in ischemic injury [72]. Of the three unique BZ markers, *Sprr1a* (small proline rich protein 1 A) has an expression pattern that follows the trajectory of scar resolution. Evaluation of *Sprr1a* expression via transcriptomics data revealed prominent enrichment within the BZ at 3 dpi, which decreases over time (Figure 7D). Consistent with spatial transcriptomics data, immunofluorescence defined the prominent expression of *Sprr1a* in BZ cardiomyocytes at 3 dpi, which subsided over time and was nearly undetectable by 21 dpi (Figure 7E). Although some Sprr1a protein was visualized in the distal (remote) zone near the base of the heart, nearly all of the protein expression was localized within immature CM at the BZ. Taken together, these results highlight the efficacy of spatial transcriptomics in defining genes with spatially restricted expression patterns at near single-cell resolution in an unbiased manner. This study also identifies regenerative transcriptional programs, and defines the BZ of the regenerating heart that expresses potentially cardio-restorative factors such as *Sprr1a*.

## 4. Discussion

The neonatal mammalian heart, similar to adult zebrafish, possesses a remarkable capacity to regenerate, which includes the transient formation of a fibrotic scar and the generation of new CM [5,19,38]. Here we conducted bulk and spatial RNA-sequencing to interrogate scar resolution during cardiac regeneration. Spatial transcriptomics revealed a dynamic phenotypic response at near single-cell resolution. The resolving scar transitions through an inflammatory to a secretory phenotype, ultimately undergoing metabolic reprogramming and acquiring a striated muscle phenotype as the scar resolves and becomes populated with immature CM (Figure 7F). This study also found that resident cardiac fibroblasts transition from a proliferative to a secretory phenotype as the heart matures and loses regenerative potential, even in the absence of injury. We also identified extracardiac fibro-lipogenic tissue harboring individual CM, indicative of damaged pericardium, surgical adhesions, incompletely removed apex, or possibly scar and CM extrusion through a disrupted epicardium. Finally, we defined the border zone of the healing cardiac apex, which is enriched in immature and potentially regenerative CM factors, including *Sprr1a*. Taken together, our study highlights the power of spatial transcriptomics in defining spatially and temporally resolved changes in tissue phenotype, providing mechanistic insight into the process of tissue repair and regeneration.

Bulk RNA sequencing of genetically labeled TCF21-lineage fibroblasts collected from hearts injured within (at P2) and beyond (at P10) the regenerative window revealed a correlation of fibroblast phenotype with postnatal age. Fibroblasts display a proliferative phenotype within the early postnatal regenerative period, consistent with the highly proliferative nature of fibroblasts during this period of rapid heart growth [73,74]. In contrast, resident fibroblasts acquire a secretory phenotype in the second week of life, even in the absence of a cardiac insult. Thus, as the heart matures and loses regenerative potential, CM become post-mitotic and resident cardiac fibroblasts become primed for ECM deposition, which displays a striking temporal correlation between the CM and CF phenotypes. Cardiac fibroblasts influence the CM phenotype by supporting fetal and neonatal CM proliferation and maturation [19,75]. It is interesting to speculate that CM may stimulate the secretory fibroblast phenotype to facilitate scar formation in the absence of a regenerative response.

While this RNA-sequencing approach revealed postnatal age-dependent fibroblast phenotypic changes, we did not detect a significant transcriptional response to apical resection injury. We suspect that transcriptional changes in a small subset of injury responsive fibroblasts become obscured by the large number of fibroblasts residing in remote tissue. We therefore employed spatial transcriptomics to assess transcriptional changes that occur during neonatal cardiac regeneration. This powerful method utilizes a 10x Visium slide containing a spotted array of spatially barcoded mRNA-capturing oligos. Following tissue permeabilization, mRNA capture, library preparation, and sequencing, gene expression is mapped back to a spot of origin on the histological specimen. Unbiased clustering defined unique transcriptional identities within the cardiac chambers. Indeed, spatial transcriptomics differentiated between the left and right chambers, compact and trabecular myocardium, and defined the epicardium, endocardium, outflow tract, and valves. It is important to note that 10x Visium is currently unable to establish transcriptional signatures at true single-cell resolution, which would be necessary to define the full contributions of various cell types that are responsible for cardiac repair. Currently, integration of spatial transcriptomics with scRNA-seq datasets are certainly necessary to realize the full potential of this technology. Despite this limitation, our study also provides a detailed and spatially resolved characterization of postnatal cardiac maturation, which includes unique maturation programs within the right atrium, trabecular myocardium, and compact ventricular myocardium. While the transcriptional identities revealed a unique response to postnatal growth, immature myocardial tissue was generally enriched in factors related to cell division and fetal CM factors such as *Nppa* and *Ankrd1* [71,76,77], which are also associated with cardiomyopathy [69,70]. In contrast, oxidative metabolism emerged as a biological pathway enriched in the maturing myocardium, indicative of the transition from glycolysis to fatty acid oxidation as a primary energy source.

Importantly, while the theoretical resolution of spatial transcriptomics is currently ~10 cells, unbiased transcriptional clustering effectively resolved the single layer of epicardial cells on the heart’s surface. Of note, we observed considerable epicardial thickening at 14 dpi coinciding with the transition to a secretory fibroblast phenotype, which is a hallmark of the injury response in the adult heart. The epicardium is essential to heart repair in zebrafish and neonatal mice [67,78], and is an important source of mitogenic factors and pro-regenerative ECM [67,78,79,80,81]. Work from our lab and others have also established the epicardium as a source of paracrine factors that guide developmental coronary vessel patterning [82,83,84,85]. Importantly, epicardium-driven revascularization is essential for mouse and zebrafish heart regeneration [21,34]. While the epicardium is also a source of paracrine factors after ischemic injury in the adult mouse, physiological levels are insufficient to support meaningful cardiac repair [68]. Indeed, the pro-angiogenic factor VEGFa declines with age, and restoring VEGF activity counteracts aging-related tissue dysfunction [86]. Thus, our newly developed dataset provides a unique opportunity to identify the spatially restricted factors that support CM proliferation and revascularization.

Fibroblast activation, ECM deposition, and scar formation are critical and transient components of regenerative cardiac repair [20]. In zebrafish, scar resolution is associated with the reversion of fibroblasts to a more quiescent phenotype [19]. However, in the neonatal mouse heart, cardiac injury is initially associated with a burst of fibroblast proliferation, and the process of scar resolution has not been well described. Interestingly, spatial transcriptomics identified the apical emergence of extra-cardiac tissue as the intracardiac fibrotic scar resolves. This extracardiac tissue initially forms a contiguous structure with the intracardiac scar that is transcriptionally defined as a single tissue type crossing a disruption in the epicardial layer. By 21 dpi the extracardiac tissue is a distinct structure and acquires a lipogenic gene profile. Immunostaining confirmed the consistent emergence of perilipin and PDGFR-α positive extra-cardiac tissue that harbors individual CM. It is likely that apical extracardiac tissue represents damaged lipogenic pericardial tissue or fibrotic surgery adhesion, although cardiac Troponin I + CM would not be expected in a surgical adhesion. Epicardium disruption is repaired in zebrafish via collective cell migration from the base to the cardiac apex, potentially allowing for a transient window for scar extrusion [87]. It is therefore possible that the scar does not entirely resolve within the regenerating postnatal mouse heart, but is at least partially extruded along with immature CM. It will be interesting to establish the identity of extracardiac tissue, and the potential biomechanical forces that drive scar extrusion.

The comparison of spatial transcriptomics and bulk RNA-seq data from isolated CF also revealed a biphasic deposition of ECM in the regenerating heart, which was confirmed by CHP staining. The initial collagen deposition occurs at 3 dpi, corresponding to the inflammatory phase. Since macrophages are essential for cardiac regeneration [16], and previous studies have described the deposition of collagen by macrophages in the regenerating heart [17], their importance may reflect involvement in the initial fibrotic response. Later in the injury response, collagen deposition temporally corresponds with the acquisition of the fibroblast secretory phenotype, which is likely responsible for the second phase of ECM deposition. A more detailed analysis of the resolving intracardiac scar also revealed five spatially and temporally distinct transcriptional profiles, including a border zone. Given its unique position between healthy and injured cardiac tissue, the border zone is an important site of active cardiac regeneration and scar resolution, previously characterized as a zone of metabolic reprogramming and immature CM repopulation [11,27]. Our study revealed an enrichment of immature CM markers, and a transient upregulation of *Sprr1a*. Sprr1a was previously correlated with a cardioprotective phenotype [72], therefore, assessing whether *Sprr1a* and other BZ-restricted factors contribute to cardiac repair will be of great interest.

In summary, our proof-of-concept study validates the efficacy of unbiased spatially resolved transcriptomics in defining gene alterations that may support heart regeneration and repair. This study reveals a dynamic response to cardiac insult, represented by the initiation of a fibro-inflammatory response, that transitions from a proliferative to a secretory phenotype during scar resolution. Importantly, spatial transcriptomics defined the border zone of regenerating heart tissue, which will facilitate the identification of pro-regenerative and anti-fibrotic mechanisms that may advance novel therapeutic approaches for cardiac repair.

## Figures and Tables

**Table 1:** Antibody concentrations.

Antyibody	Vendor/Cat No.	Concentration
Mouse anti-Cardiac Troponin I (C-4)	Santa Cruz sc133117	1/30
Rabbit anti-ERG	Abcam ab92513	1/100
Rabbit anti-GFP	Torrey Pines Biolabs TP401	1/200
Goat anti-PDGFR-alpha	R&D Systems AF1062	1/200
Rabbit anti-Perilipin	Abcam ab3526	1/100
Rabbit anti-Sprr1a	Abcam ab125374	1/100
Secondary & Tertiary Antibodies		
Donkey anti-mouse 647	Jackson ImmunoResearch Laboratories #715-605-150	1/100
Donkey anti-rabbit Biotin	Jackson ImmunoResearch Laboratories #711-065-152	1/500
Bovine anti-goat HRP	Jackson ImmunoResearch Laboratories #805-035-180	1/200
Tyramide Cy3	Perkin Elmer NEL744001KT	1/200
Streptavidin 488	LifeTech S11223	1/100
Streptavidin 555	LifeTech S21381	1/100
DAPI	Invitrogen D1306	1/10,000

## Data Availability

Bulk RNA-sequencing and spatial transcriptomics data are available in the Gene Expression Omnibus (GEO), as of 20 December 2021. Bulk RNA-sequencing: https://www.ncbi.nlm.nih.gov/geo/query/acc.cgi?acc=GSE188844 Spatial transcriptomics: https://www.ncbi.nlm.nih.gov/geo/query/acc.cgi?acc=GSE188888.

## Supplementary Materials

The following are available online at: https://www.mdpi.com/article/10.3390/jcdd9010001/s1, Figure S1: Biological processes that are enriched in fibroblasts. Figure S2: Anatomical annotation of spatial transcriptomics spots in postnatal heart; Figure S3: Spatially resolved expression in the postnatal ascending aorta and valves; Figure S4: Spatially resolved expression in the postnatal atria; Figure S5: Spatially resolved expression in the postnatal ventricles; Figure S6: Pseudotime analysis of the regenerative scar; Figure S7: Biological gene ontology programs were identified that correspond with regenerative scar pseudotime state; Figure S8: Pseudotime analysis of the intracardiac resolving scar; Figure S9: Biological gene ontology programs were interrogated that correspond with intracardiac resolving scar; Table S1: List of differentially expressed genes in sham vs resection at P2 plus 3 days; Table S2: List of differentially expressed genes in sham vs resection at P10 plus 3 days; Table S3: List of differentially expressed genes at baseline P2 versus P10; Table S4: List of genes that are enriched in particular clusters, compared to all the other clusters.
