# Supplementary material for: Characterizing Neonatal Heart Maturation, Regeneration, and Scar Resolution Using Spatial Transcriptomics
Source: J Cardiovasc Dev Dis. 2021 Dec 21;9(1):1. doi: 10.3390/jcdd9010001 (PMC8779463; doi:10.3390/jcdd9010001)
Supplement: Supplementary file 1 [file jcdd-09-00001-s001.zip › Misra_Supplemental files/Misra_SupplementaryFigures.pdf]

Supplementary Figures

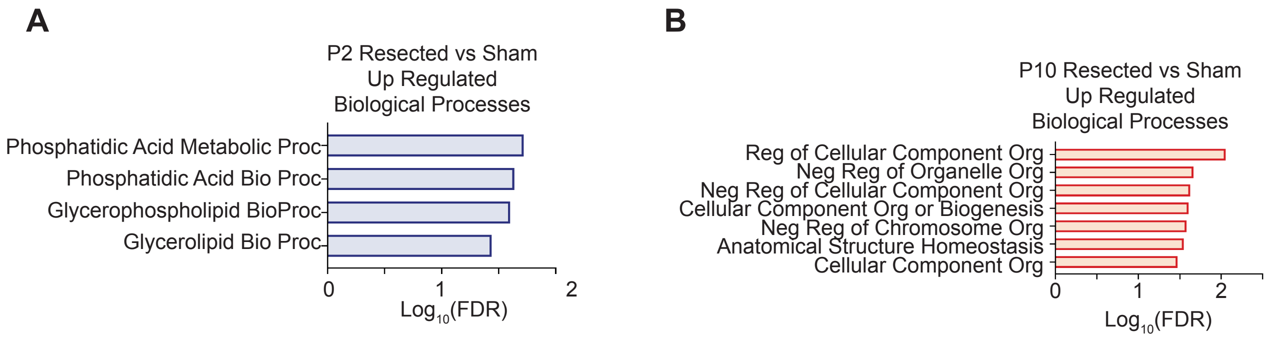

Fig. S1. *Biological processes that are enriched in fibroblasts.* **A, B.** Differentially expressed genes in fibroblasts isolated 3 days after apical resection or sham surgery revealed Biological processes that are enriched upon resection surgery at (A) and (B) P10.

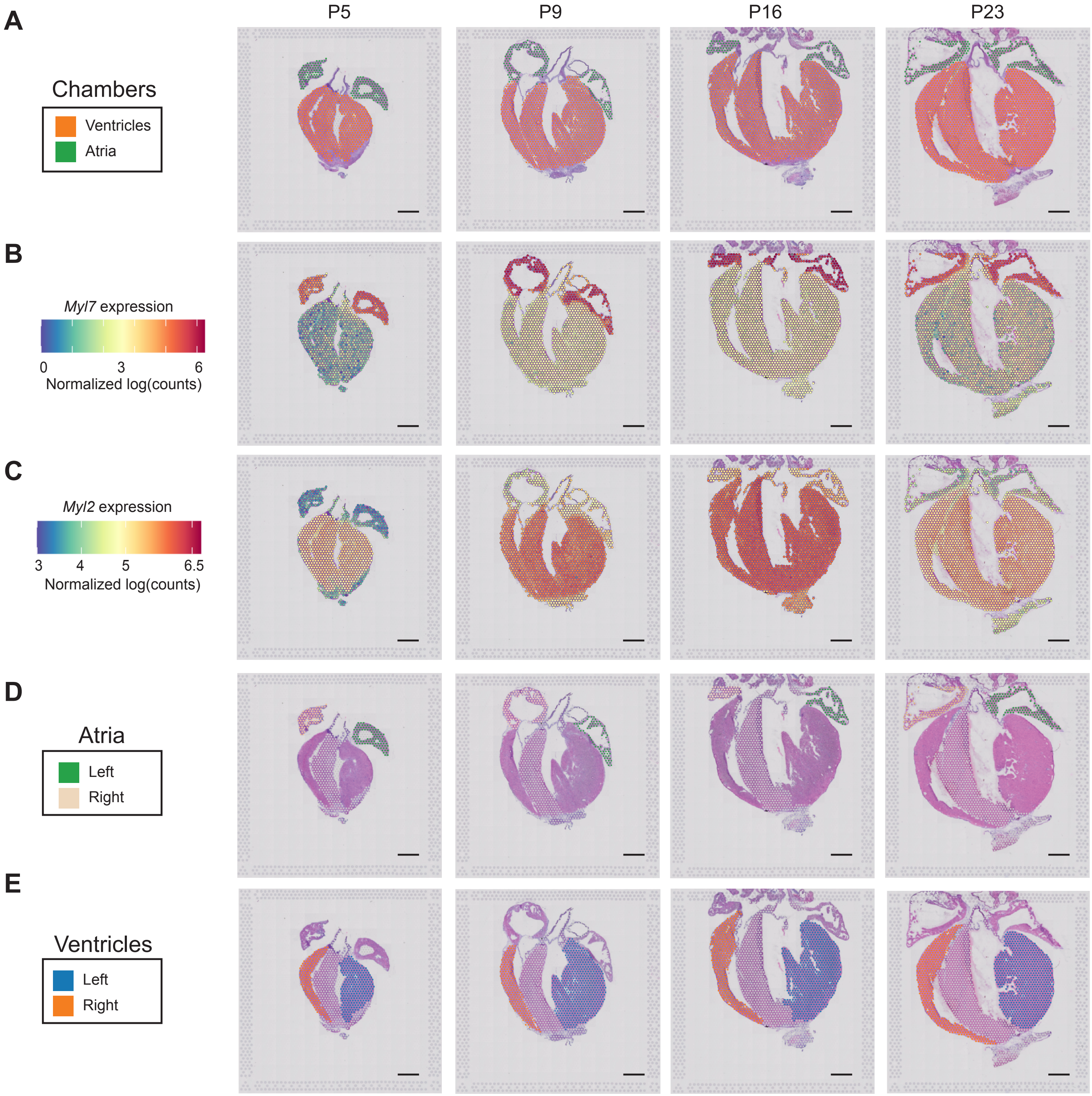

Fig. S2. *Anatomical annotation of spatial transcriptomics spots in postnatal heart.* **A.** Loupe Browser was used to assign each spot an identity as (A) ventricle or atria. **B, C**. Spatially resolved expression of (B) Myl7 and (C) Myl2 reveals an enrichment in the atria and ventricles, respectively. **D, E.** Loupe Browser was used to assign each spot an identity as (D) left versus right atria; or (E) left versus right ventricle.

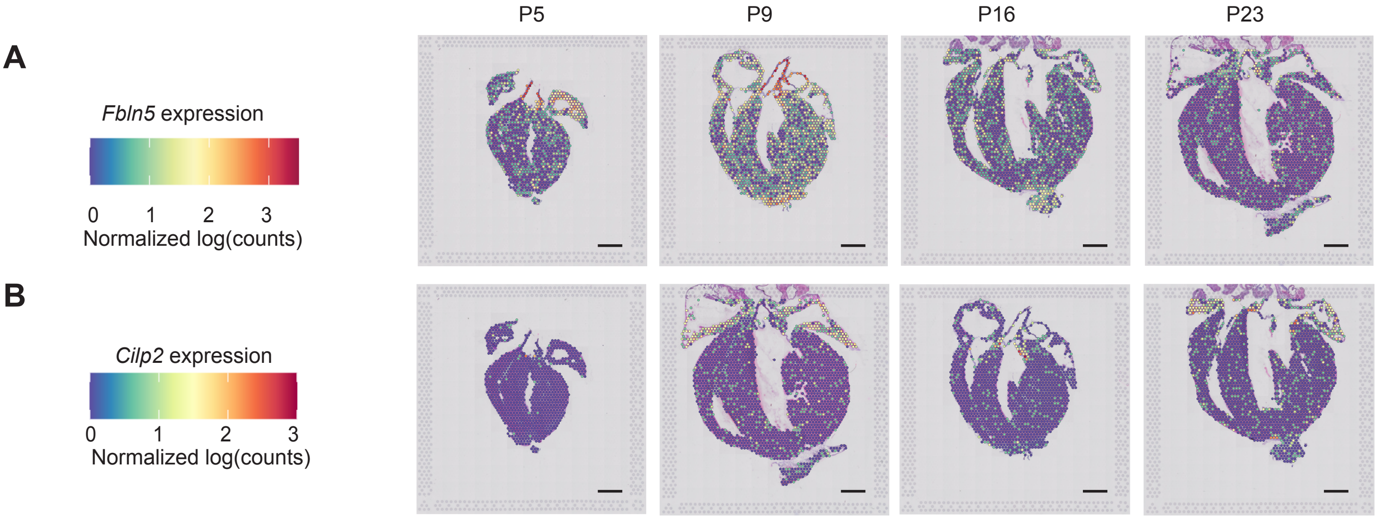

C

GO: Biological Process	DEGs/Total	p-val	p-val (adj)
extracellular matrix organization (GO:0030198)	55/229	3.57E-40	9.28E-37
neutrophil degranulation (GO:0043312)	64/479	1.34E-30	1.74E-27
neutrophil activation involved in immune response (GO:0002283)	64/483	2.18E-30	1.89E-27
neutrophil mediated immunity (GO:0002446)	64/487	3.55E-30	2.30E-27
platelet degranulation (GO:0002576)	28/124	3.88E-20	2.01E-17

Fig. S3. *Spatially resolved expression in the postnatal ascending aorta and valves.* **A, B.** Spatially resolved expression of (A) *Fbln5* and (B) *Cilp2* expression reveals an enrichment in the ascending aorta, valves, and atria. **C.** Biological Processes enriched in ascending aorta and valve spots (cluster 7).

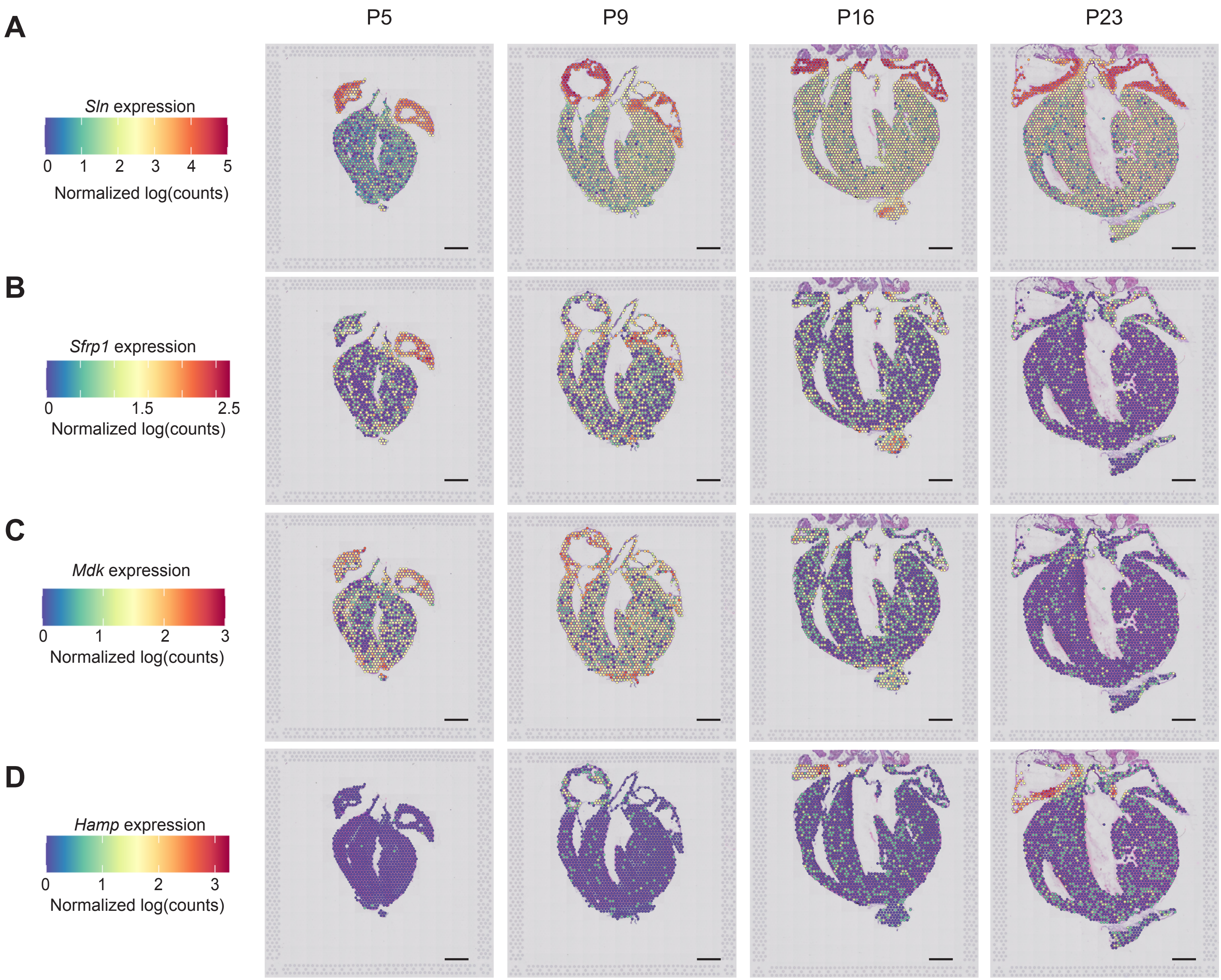

Fig. S4. *Spatially resolved expression in the postnatal atria.* **A-D.** Spatially resolved expression of *Sln*, *Sfrp1*, *Mdk*, and *Hamp* in the postnatal atria. (A) Note Sln expression throughout both atria at all timepoints; (B, C) *Sfrp1* and *Mdk* expression localized to the left and right atrium, respectively, in the early postnatal heart; and (D) *Hamp* expression localized to the right atrium as the heart matures.

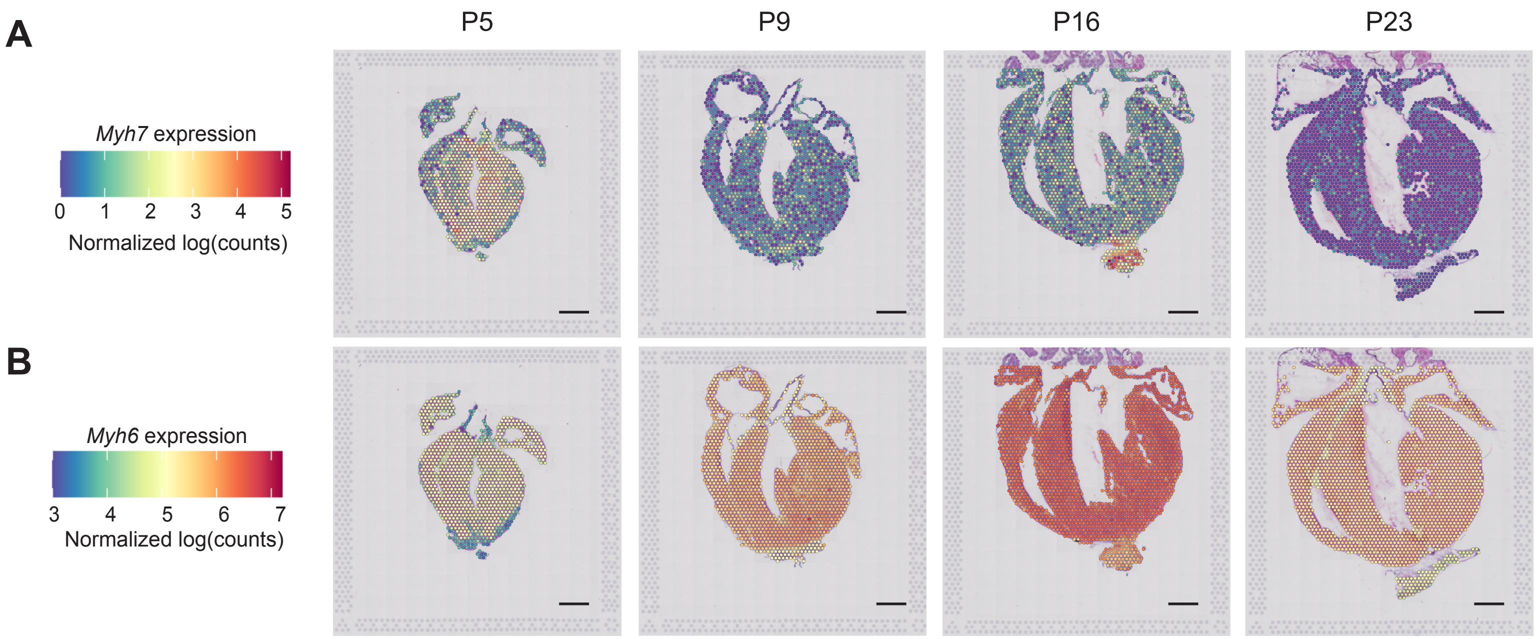

Fig. S5. *Spatially resolved expression in the postnatal ventricles.* A, B. Spatially resolved expression of *Myh7* and *Myh6* in the postnatal ventricles. (A) Note the enrichment of *Myh7* in the early postnatal timepoints, and (B) the enrichment of *Myh6* as the heart matures.

A

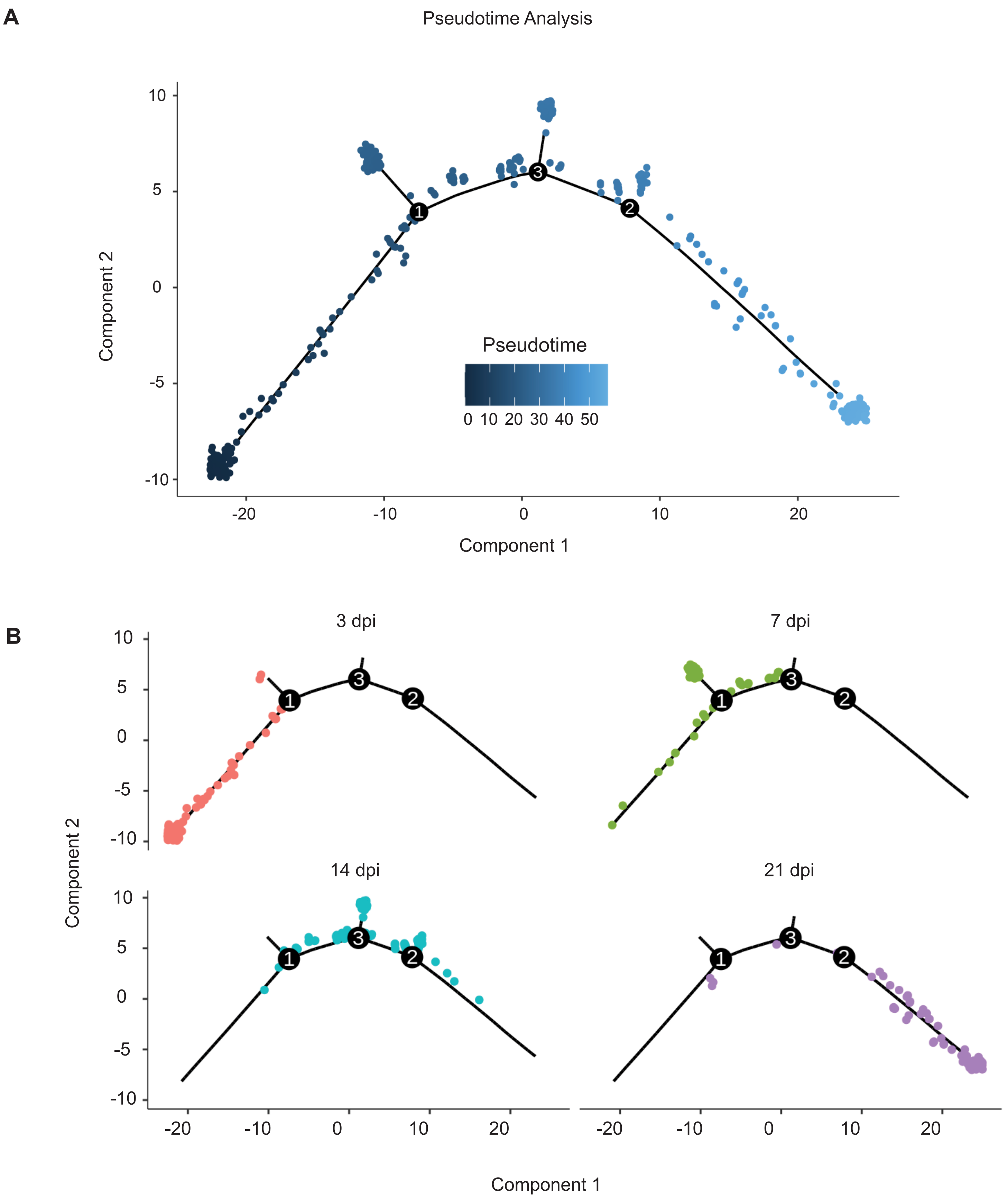

Fig. S6. *Pseudotime analysis of the regenerative scar.* **A.** Monocle was used to establish developmental pseudotime trajectory of all spots correlating with scar identity. **B.** Developmental trajectory was plotted as a function of days post injury (dpi).

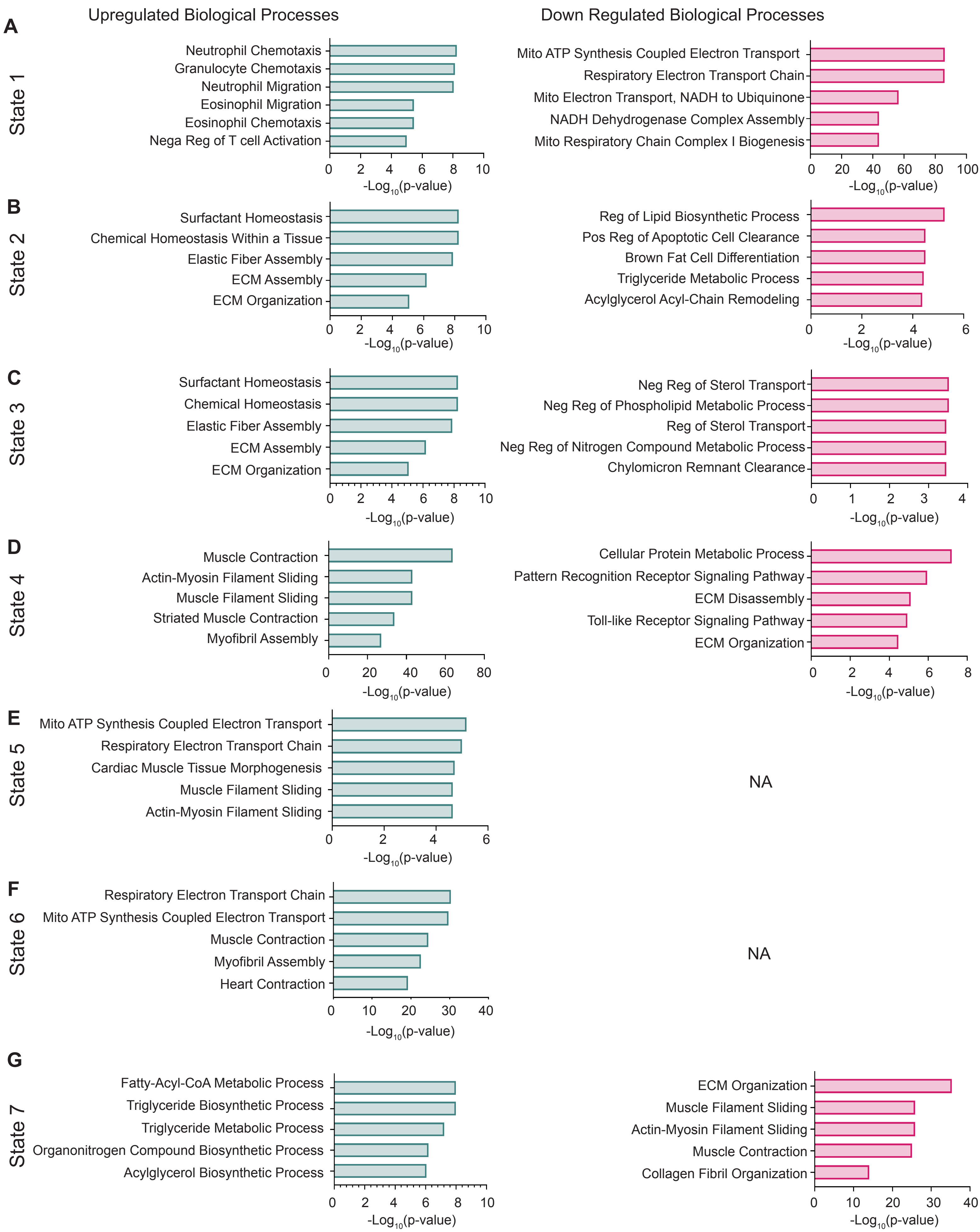

Fig. S7. Biological gene ontology programs were identifed that correspond with regenerative scar pseudotime state.

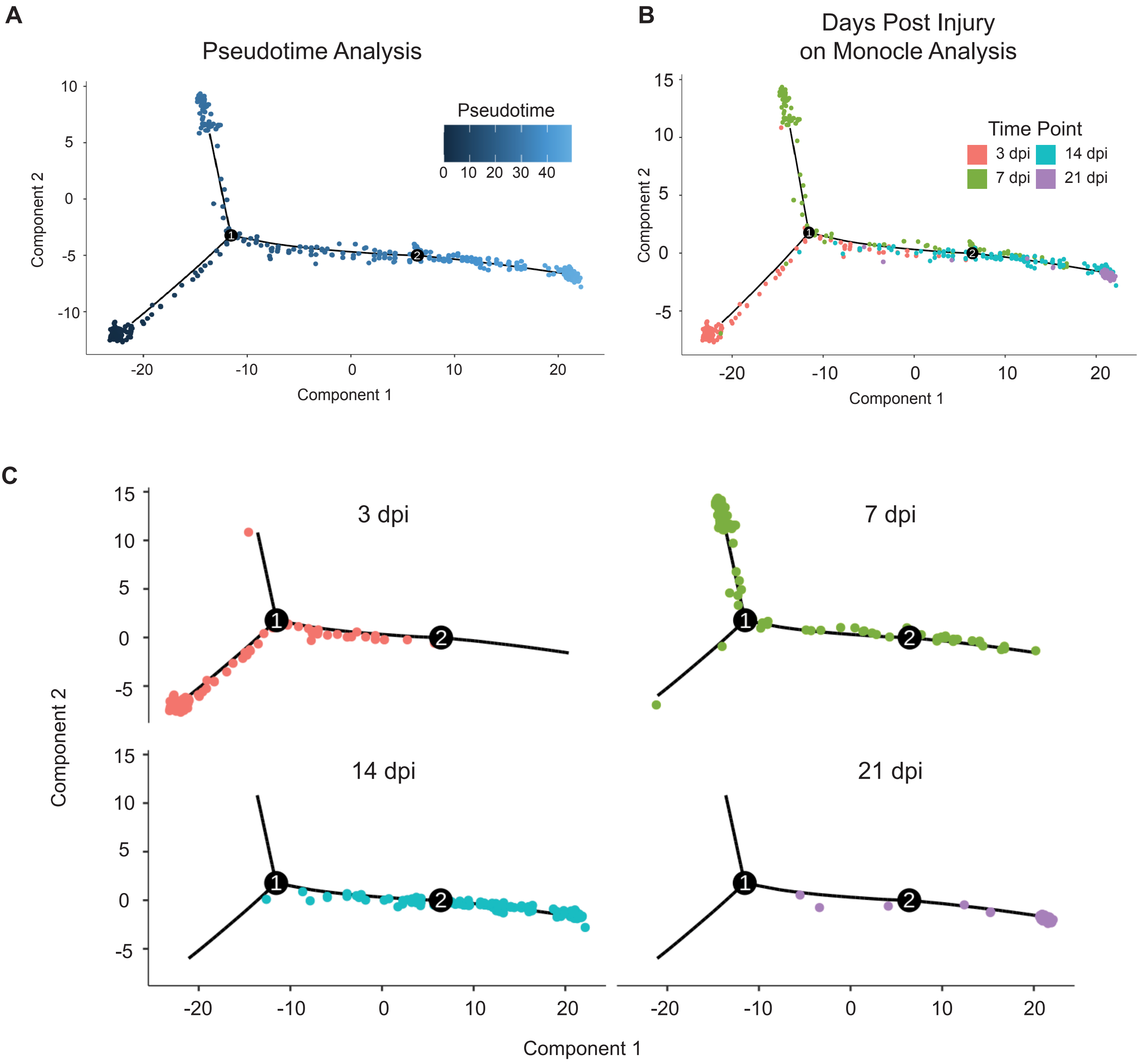

Fig. S8. *Pseudotime analysis of the intracardiac resolving scar.* **A.** Monocle was used to establish developmental pseudotime trajectory of all spots correlating with scar identity. **B, C.** Developmental trajectory was plotted as a function of days post injury (dpi).

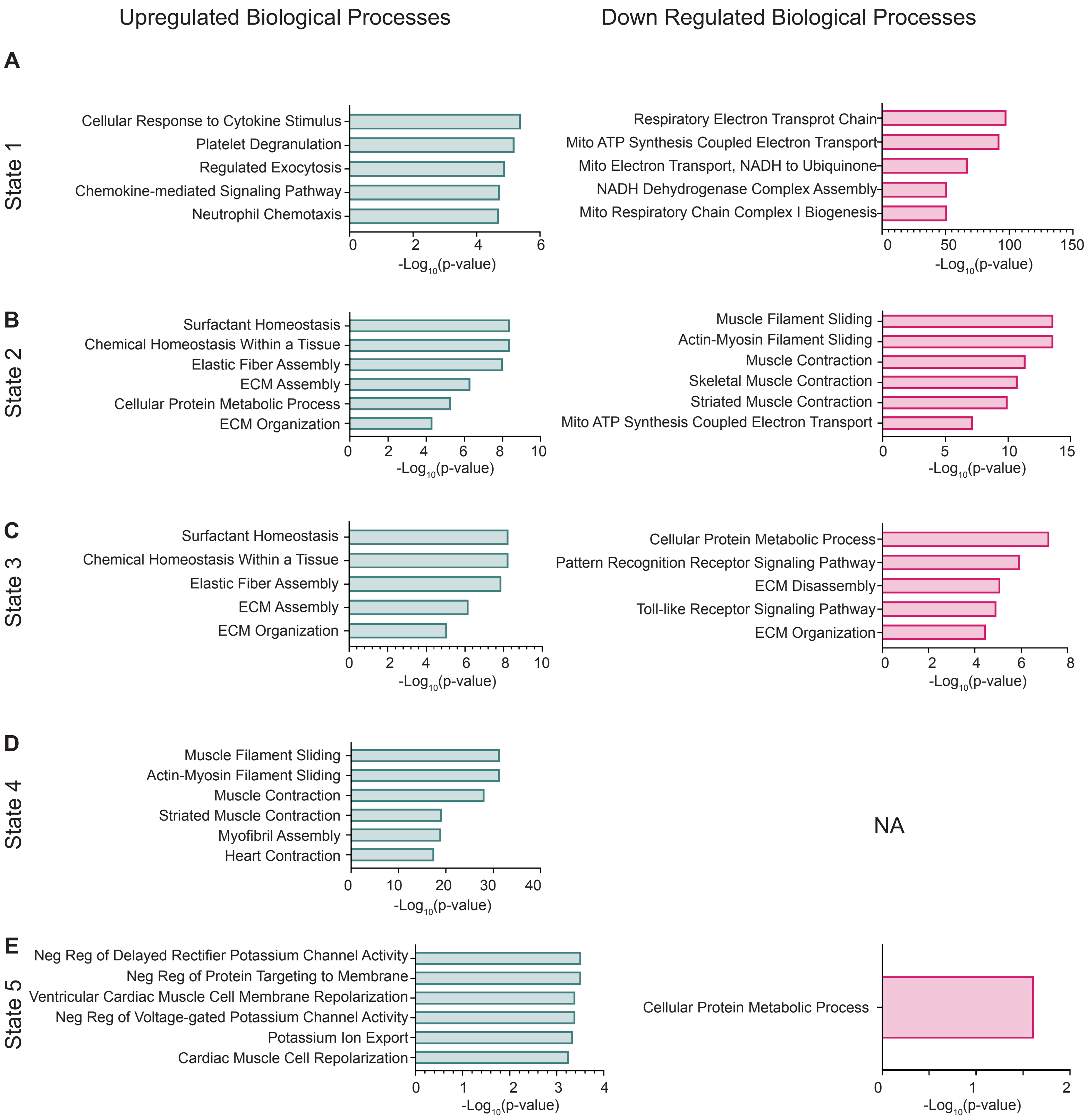

Fig. S9. Biological gene ontology programs were interrogated that correspond with intracardiac resolving scar.
